# Targeting Galectin 3 illuminates its contributions to the pathology of uterine serous carcinoma

**DOI:** 10.1038/s41416-024-02621-x

**Published:** 2024-03-04

**Authors:** Yusuke Matoba, Dominique T. Zarrella, Venkatesh Pooladanda, Maryam Azimi Mohammadabadi, Eugene Kim, Shaan Kumar, Mengyao Xu, Xingping Qin, Lauren J Ray, Kyle M. Devins, Raj Kumar, Artem Kononenko, Eric Eisenhauer, Irva E. Veillard, Wataru Yamagami, Sarah J. Hill, Kristopher A. Sarosiek, Oladapo O. Yeku, David R. Spriggs, Bo R. Rueda

**Affiliations:** 1 Vincent Center for Reproductive Biology, Department of Obstetrics and Gynecology, Massachusetts General Hospital, Boston, MA 02114 USA; 2 Harvard Medical School, Boston, MA 02115 USA; 3 Department of Obstetrics and Gynecology, Keio University School of Medicine, Shinjuku-ku, Tokyo, 160-8582 Japan; 4 Harvard T.H. Chan School of Public Health, Boston, MA 02114 USA; 5 Department of Pathology, Massachusetts General Hospital, Boston, MA 02114 USA; 6 Division Gynecologic Oncology, Department of Obstetrics and Gynecology, Massachusetts General Hospital, Boston, MA 02114 USA; 7 Department of Medical Oncology and Division of Molecular and Cellular Oncology, Dana-Farber Cancer Institute, Boston, MA 02215 USA; 8 Division of Hematology-Oncology, Massachusetts General Hospital, Boston, MA 02114 USA; 9 Department of Medicine, Massachusetts General Hospital, Boston, MA 02114 USA

**Keywords:** Endometrial cancer, Cancer stem cells, Cell migration, Cancer microenvironment, Cell growth

## Abstract

**Background:**

Uterine serous cancer (USC) comprises around 10% of all uterine cancers. However, USC accounts for approximately 40% of uterine cancer deaths, which is attributed to tumor aggressiveness and limited effective treatment. Galectin 3 (Gal3) has been implicated in promoting aggressive features in some malignancies. However, Gal3’s role in promoting USC pathology is lacking.

**Methods:**

We explored the relationship between *LGALS3* levels and prognosis in USC patients using TCGA database, and examined the association between Gal3 levels in primary USC tumors and clinical-pathological features. CRISPR/Cas9-mediated Gal3-knockout (KO) and GB1107, inhibitor of Gal3, were employed to evaluate Gal3’s impact on cell function.

**Results:**

TCGA analysis revealed a worse prognosis for USC patients with high *LGALS3*. Patients with no-to-low Gal3 expression in primary tumors exhibited reduced clinical-pathological tumor progression. Gal3-KO and GB1107 reduced cell proliferation, stemness, adhesion, migration, and or invasion properties of USC lines. Furthermore, Gal3-positive conditioned media (CM) stimulated vascular tubal formation and branching and transition of fibroblast to cancer-associated fibroblast compared to Gal3-negative CM. Xenograft models emphasized the significance of Gal3 loss with fewer and smaller tumors compared to controls. Moreover, GB1107 impeded the growth of USC patient-derived organoids.

**Conclusion:**

These findings suggest inhibiting Gal3 may benefit USC patients.

## Introduction

Endometrial cancer is the most common cancer of the female reproductive organs. It was estimated there would be roughly 66,200 new cases reported annually in the United States in 2022 [1]. Eighty-five percent of endometrial cancers are histologically endometrioid carcinoma. However, uterine serous carcinoma (USC), which accounts for about 5-10% of all uterine cancers, has a poor prognosis [2, 3]. USC has a high rate of metastatic lesions, even when the primary lesion is contained locally (i.e., in a polyp). Patients with USC have a higher risk of recurrence and shorter overall survival than endometrioid carcinoma at the same stage. While fewer in total number, USC accounts for about 40% of all cancer deaths from uterine cancer [2]. While white women experience a higher incidence of endometrial cancer overall, mortality disproportionally affects black women [4]. It has been reported that USC accounts for 30% of all endometrial cancer in black women [5] and may contribute to the increase in endometrial cancer deaths among them [6, 7]. Unfortunately, despite adjuvant therapy, patients with USC develop systemic relapses [8]. Overcoming this aggressive tumor histology is an urgent clinical issue. This can only be done by better defining the factors and their mechanisms contributing to their aggressive nature.

A family of glycan recognition proteins called galectins have been reported to be involved in cancer progression and metastasis [9]. Galectins are a family of endogenous lectins defined as soluble small β-galactoside binding proteins [10]. Based on the structure and number of their carbohydrate recognition domains (CRDs), galectins are classified into three types: homodimers with one CRD, tandem repeats with two CRDs, and chimeric types in which one CRD is bound to a non-lectin domain and can form a multimeric structure [11]. Galectin 3 (Gal3) is involved in promoting proliferation and metastasis [12], stimulating angiogenesis [13], mediating immune suppression [12], and chemotherapeutic resistance [14] in various carcinomas. Our previous work highlighted the effectiveness of Gal3 inhibition in disrupting Mucin 16 (MUC16) induced tumor-promoting properties and its reduction improved overall survival in breast and ovarian xenograft models [15].

Currently, there is no consensus regarding the relative expression and functional role of Gal3 in uterine cancer. What is known, is primarily from immunohistochemical assessments and these findings differ, often to the point of contrasting [16–18]. Our studies demonstrating the impact of Gal3 in tumor progression in highly aggressive ovarian cancer [15] led us to consider whether Gal3 contributed to the aggressive features of USC. Thus, we investigated the expression and more importantly the functional contribution of Gal3 in tumor promoting properties using in vitro and in vivo pre-clinical models of USC.

## Materials and Methods

Unless indicated, detailed methods, methods for supplementary data and lists of reagents, equipment, and searches are provided in supplementary files to ensure repeatability by others.

### Gal3 expression and its relation to clinical features

#### The Cancer Genome Atlas (TCGA) database analysis

The expression levels of LGALS3 and its correlation with progression-free survival (PFS) and overall survival (OS) were evaluated. Patients exhibiting LGALS3 expression z-scores greater than 1 were categorized as LGALS3 high group, while those with LGALS3 expression z-scores lower than −1 were assigned to the LGALS3 low group. A comparison was made between the LGALS3 high and low groups for patients with USC, respectively.

#### Clinical samples

Paraffin blocks representing primary surgical cases from patients with a diagnosis of USC were retrieved from either the Massachusetts General Hospital (MGH) Department of Pathology archives and from the Vincent Center for Reproductive Biology (VCRB)-MGH Gyn Repository. The samples were collected from cases between in 1994 and 2014 based on availability and quality of the paraffin blocks, viable tumor and available clinical data. All diagnosis were verified. All samples were obtained under Institutional Review Board approved protocol (MGB022P003117 and DFHCC07-049). Patient-derived USC organoids (PDOs) were provided by Dr. Sarah Hill, Dana Farber Cancer Institute.

#### Evaluation of Gal3 expression in primary tumors and its relationship to clinic-pathological features

Paraffin blocks representing the primary tumor from each patient were serially sectioned and stained with hematoxylin and eosin (H&E) and probed with anti-Gal3 antibody as described [19]. The anti-Gal3 antibody was validated using Gal3 positive and Gal3 knockout (Gal3-KO) cell lines (Supplementary Fig. S1a, b). Two certified pathologists blinded to the original pathology report and diagnosis, scored Gal3 expression by the percentage of Gal3-positive tumor cells as previously reported [20]. Their findings were correlated with clinical parameters.

### Cell lines and organoids culture

The ARK1 and ARK2 USC cell lines were provided by Dr. A. Santin (Yale University, New Haven, CT, USA) [21, 22] and cultured as described [19]. The ovarian SKOV3 cancer cells were maintained as described [23]. Human umbilical vein endothelial cells (HUVECs) were purchased from LONZA and were maintained in EBM-2 medium supplemented with Endothelial Cell Growth Medium 2 Supplement Pack. IMR90 cells, normal fibroblast cells, were kindly provided by Dr. Cesar Castro (MGH, Boston, MA, USA). This cell line was maintained in Eagle’s Minimum Essential Medium (EMEM) supplemented with 10% Fetal bovine serum (FBS) and 1% penicillin-streptomycin. All cells were maintained at 37 °C at 5% CO_2_. The cell lines were confirmed by short tandem repeat analysis and tested negative for mycoplasma prior to any assay by the MycoAlert Mycoplasma Detection Kit (Lonza). The PDOs were maintained as previously described [24].

### Generation of Gal3-KO cells and knockdown cells

Oligonucleotides targeting LGALS3 were synthesized, annealed, and ligated into the single guide RNA scaffold of the LentiCRISPRv2 via the BsmBI sites, according to the method previously described [25]. Two different oligo pairs were chosen for construct synthesis targeting the following sequences in exon 3 (sgRNA1) or 4 (sgRNA2) of LGALS3: sgRNA1 GTCTACCCAGGGCCACCCAG and sgRNA2 GCTGATAACAATTCTGGGCA. Successful construct generation was verified by Sanger sequencing of the sgRNA region. Transfections of ARK1 and ARK2 cells were performed using Lipofectamine 2000 with co-transfection of pLenti-C-mGFP-P2A-Puro Lentiviral Gene Expression Vector to detect the cells which were transfected. Gal3-KO cells obtained using sgRNA1 in ARK1 and sgRNA2 in ARK2 cells and Gal3 control cells (Gal3-CTRL) generated using the same method without gRNA were used in subsequent experiments. The SKOV3 Gal3 knockdown (Gal3-KD) cell line was generated utilizing the shRNA system as described [26].

### Conditioned Media (CM) for add back experiments

ARK2 Gal3-CTRL and Gal3-KO cells were counted and plated at concentrations estimated to reach 70-80% confluence after three days. The day after plating, the complete media was changed out to media containing 10% or 2% FBS. After 48 h, the conditioned medium was collected and centrifuged at 3500 rpm for 5 min. The supernatant was filtered with a 0.2 μm surfactant free cellulose acetate (SFCA) filter and applied to the further experiments.

### Assessment of cell viability and proliferation

Cell counts: 0.1 × 10^6^ cells/well were seeded on the 6-well plates with complete media. Cell counts were determined with a TC20 Automated Cell Counter (Bio-Rad Inc) at the indicated time points (time zero = overnight incubation, 24, 48, and 72 h after overnight incubation) in triplicate. Dead cells were excluded using the trypan blue staining.

#### Cell cycle assay

A cell cycle assay was performed using the Cell Cycle Phase Determination Kit (Cayman), following the kit’s instructions. Briefly, after 24 h of serum-free starvation treatment, each well was changed to 10% FBS-containing medium, and after 6 h, the cells were subjected to cell cycle assay. At least 20,000 events for each end point measure were evaluated. The FlowJo™ cell cycle analysis module (Dean-Jett-Fox model) was used to analyze the percentage of population for each of the subG1, G0/G1 phase, S phase, and G2/M phase.

#### MTT assay to measure the chemosensitivity

Cells were harvested at sub-confluency and plated in 96-well plates at 1000 cells per well in 100 μL of complete media. After overnight incubation, 200 μL of the complete media containing increasing concentrations of carboplatin (0, 1–20 µM) was added to each well. 72 h after adding the media, cell viability was assessed by MTT assay as described [27].

#### BH3 profiling

To ascertain the degree of involvement of Gal3 in cell priming towards apoptosis, we conducted BH3 profiling as described previously [28].

#### Investigation for the impact of loss/inhibition of Gal3 on EGFR signaling

ARK1 and ARK2 cells were seeded and cultured in a complete media with 10% FBS. When cells reached sub confluence, the media was replaced with complete media containing 10 µM of GB1107, a Gal3 small molecule inhibitor (SMI). The cells were cultured for 12 or 72 h, and cell lysates were utilized for immunoblot analysis.

### Assessment of cancer stem cell properties

#### Colony and sphere-forming assays

Colony and sphere-forming assays were conducted as previously described [29]. The colony-forming assay was repeated using 10% FBS CM from ARK2 Gal3-CTRL or ARK2 Gal3-KO mixed with the complete media (1:1 ratio) to assess the add-back effect.

#### Impact of pharmacologic inhibition of Gal3 on Notch1 signaling

ARK1 and ARK2 cells were seeded and cultured in the organoid culture condition [24]. Cells were cultured in the organoid culture media for 7 days with 10 µM of GB1107 or vehicle (DMSO), being added every other day in new media. After 7-days, the cells were collected, and the lysates were subjected to immunoblot analysis.

### Exploring the role of Gal3 in adhesion, migration, invasion, and angiogenesis

#### Fibronectin adhesion assay

The fibronectin-coated 96 well-plate was made by adding 80 μL of fibronectin solution (1 μg/mL) and incubating for 1 h at room temperature (RT). After removing the solution, the wells were rinsed with phosphate-buffered saline (PBS) three times. Ten thousand cells/well were seeded with 100 μL of complete media and incubated for 1 h at 37 °C, 5% CO_2_. After incubation, wells were rinsed gently with PBS, and cells were fixed with methanol for 15 min at RT. Cells were then stained with crystal violet for 20 min at RT. After staining, the wells were washed with dH_2_O, and images recorded using a EVOS M5000 Imaging System (Thermo Fisher Scientific) at 40× magnification. The number of adherent cells was counted by Fiji software version 2.3.0.

#### Trans-well migration and Invasion assay

Both assays were conducted as previously described with slight modifications [30, 31]. Four images at different locations were taken of each insert under 40× magnification of Nikon ECLIPSE Ni-U and the number of migrated cells was quantified using Fiji software version 2.3.0.

#### Angiogenesis invasion and Tube-forming assay using HUVECs

These assays were performed with HUVECs, which were cultured following the method described earlier. The protocols for angiogenesis invasion and tube-forming assays were carried out on HUVECs with slight modifications as described in a previous study [32]. The analysis of angiogenesis invasion assay followed the same procedure as the trans-well and invasion assays mentioned earlier. For the tube-forming assay, the data were analyzed using Wimasis image analysis software, and parameters such as the total length of tubes and the number of branch points were evaluated.

Gal3 influence on the transition of fibroblasts to cancer-associated fibroblasts (CAFs): IMR90 cells were seeded on Falcon 4 well culture slide with 10,000 cells per chamber for immunocytochemistry (ICC) or on a 6 cm dish with 150,000 cells per dish. After overnight incubation, cells were treated with a 1:1 mixture of the complete media described above and 10% FBS CM from ARK2 Gal3-CTRL or ARK2 Gal3-KO. After 48-hour incubation, treated cells were either assessed via ICC or lysates were subjected to immunoblotting.

### Loss of Gal3 effect using in vivo models and patient derived organoids

#### Animal protocols

All animal experiments were approved by the Massachusetts General Brigham Institutional Animal Care and Use Committee and performed according to the National Institutes of Health (NIH) Guide for the Care and Use of Laboratory Animals (Protocol number: 2017N000236). Female NSG (NOD scid gamma) mouse, 6–8 weeks of age, were obtained from Steele Laboratories at MGH. The number of mice used in our in vivo experiments was based on preliminary experiments conducted using these specific cell lines. The experiments did not require blinding and randomization.

#### In vivo limiting dilution tumorigenic assay

ARK1 Gal3-CTRL and KO cells were counted, aliquoted and resuspended in the mixture of 100 µL of PBS and100 µL of Cultrex Basement Membrane Extract, type 2. Then, 10, 50, 100, and 200-thousand cells were injected subcutaneously in the dorsal flank of each mouse (*n* = 4/group) using 28-gauge needle. Mice were palpated for tumor every two days to determine time to tumor onset. Mice were then weighed, and tumor growth monitored. Mice were euthanized in accordance with IACUC protocol and tumors collected, weighed, and processed. The extracted tumors were formalin-fixed and paraffin-embedded. Representative sections were subjected to H&E and IHC (Ki67, and phospho-Histone H3 (pHH3)) staining as described [19, 33].

#### Intraperitoneal (IP) tumor model

One million of ARK1 Gal3-CTRL or KO cells were injected in a volume of 200 µL PBS, IP (*n* = 4/group). Sixty days post injection the mice were euthanized. The peritoneal cavity was imaged to document disseminated lesions. Lesions were harvested, total tumor weight determined and processed as described above.

#### Investigation of Gal3 pharmacological inhibition effect using patient-derived organoids

Organoids were seeded in Matrigel on 48-well plates and cultured in organoid culture medium, containing 10 µM of GB1107 or vehicle; dimethyl sulfoxide (DMSO). The media was replenished every other day, and average area occupied by the organoids was quantified after 14 days using an Incucyte Live Cell System and automated Organoid Software Analysis Module (Essen BioScience).

### Immuno Cyto Chemistry (ICC)

Cells were fixed with 4% paraformaldehyde and permeabilized using 0.1% Triton X. The cells were then incubated with 3% bovine serum albumin at RT for 1 h. Primary or isotype control antibodies were added to wells and incubated at RT for 1 h. After rinsing with PBS and PBST, the cells were incubated with the secondary antibody for 1 h at RT in the dark. Following rinsing with PBS and PBST, the cells were stained with Phalloidin if required, at RT for 1 h. The cells were then rinsed with PBS and mounted with DAPI. Finally, the cells were imaged using the FV3000 confocal laser scanning microscope (Olympus).

### Immunoblot analysis

Immunoblot analysis was performed as previously described [19]. Details for specific antibodies, dilutions, development, imaging and quantification can be found in supplementary files.

### Data presentation and statistical analysis

All experiments were independently replicated at least three times. Data shown in graph format represent means ± SEM of combined results from all experimental replicates, whereas representative histological photomicrographs are indicated where appropriate. Statistical analyses were done with GraphPad Prism version 9.3.1. T-test was used for experiments in which one outcome or multiple independent outcomes were obtained. One-way ANOVA (analysis of variance) was employed for the comparison among multiple groups, followed by post hoc analysis using the Dunnett’s test. Two-way ANOVA was used for the experiments with multiple related outcomes, and group comparisons were performed using Šidák correction. The χ-square test was employed to analyze the contingency table. All statistical analyses were two-sided test. A *p*-value < 0.05 was considered statistically significant.

## Results

### Correlation of Gal3 and *LGALS3* and clinical characteristics

**Fig. 1:**
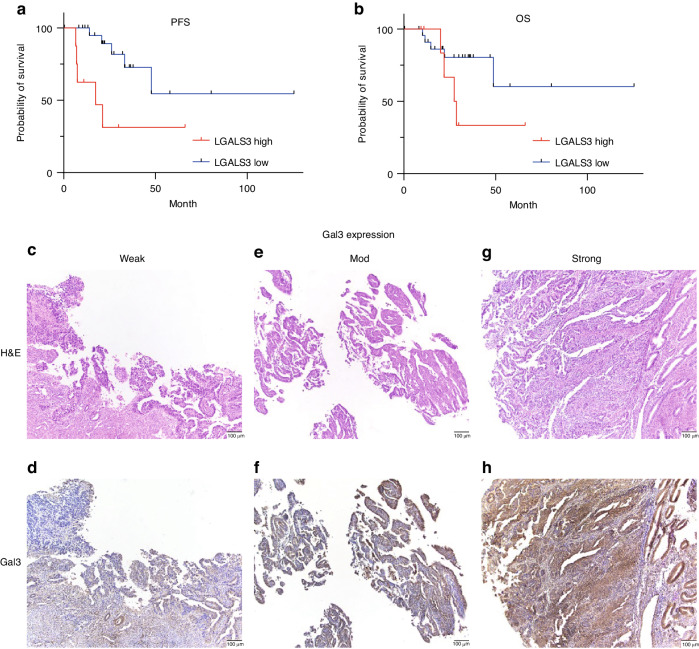
Clinical features related to *LGALS3* and Gal3 levels. **a** Kaplan-Meier curves representing PFS for patients that are *LGALS3* high or low in USC cohort (*p* = 0.0088). **b** Kaplan-Meier curves representing OS for the same patient group (ns). **c** H&E staining and **d** IHC staining of Gal3 low-expression. **e** H&E staining and **f** IHC staining of Gal3 moderate-expression. **g** H&E staining and H) IHC staining of Gal3 high-expression. All images were captured under 100 × magnification. The *p* value was computed using the log-rank test. The scale for staining was based on the percentage of Gal3-positive tumor cells as follows: low expression, in which 0–5% of tumor cells were Gal3-positive; moderate expression, in which 6–49% of tumor cells were positive; and high expression, in which 50% or more of tumor cells were positive.

The TCGA uterine cancer data set revealed patients with high levels of *LGALS3* had a significantly shorter PFS compared to those with low levels of *LGALS3*) (*p* = 0.0088) (Fig. 1a, b). The levels of Gal3 were assessed in 37 different USC cases by two pathologists and its relationship with clinical parameters was determined (summarized in Table 1). Representative Gal3 low, moderate, and high expression samples are shown in Fig. 1c–h. Patients with no or low level of Gal3 expression in their primary tumors were less likely to present with advanced stages (*p* = 0.0028), LVSI (lymphovascular space invasion) (*p* = 0.0053), cervical involvement (*p* = 0.0171), and pelvic and paraaortic lymph node metastases (pelvic: *p* = 0.0067, paraaortic: *p* = 0.0332).

**Table 1:** Relationship between Gal3 expression level and clinical pathological features.

		Galectin 3			
Total patient number: 37		Weak (*n* = 6)	Mod (*n* = 16)	Strong (*n* = 15)	*p* value
Age (Median, range)		63 y.o. (57–83)	66.5 y.o. (56–84)	69 y.o. (58–82)	0.9306
FIGO Stage**	I	6	7	4	0.0028
(*n* = 37)	II	0	1	0	
	III	0	1	6	
	IV	0	7	5	
Myometrium invasion	<50%	5	8	8	0.3507
(*n* = 37)	≥50%	1	8	7	
LVSI**	Absent	6	7	3	0.0053
(*n* = 34)	Present	0	7	11	
Serosal involvement	Absent	6	12	10	0.2734
(*n* = 37)	Present	0	4	5	
Cervical involvement*	Absent	6	10	5	0.0171
(*n* = 37)	Present	0	6	10	
Ovarian metastasis	Absent	6	11	10	0.2969
(*n* = 36)	Present	0	5	4	
Omental metastasis	Absent	4	7	8	0.1786
(*n* = 30)	Present	0	7	4	
Pelvic lymph node metastasis**	Absent	5	8	3	0.0067
(*n* = 26)	Positive	0	2	8	
Para-aortic lymph node metastasis*	Absent	3	5	1	0.0332
(*n* = 16)	Positive	0	2	5	
Distant metastasis	Absent	6	9	10	0.148
(*n* = 37)	Positive	0	7	5	
Ascites cytology	Negative	4	7	9	0.4127
(*n* = 28)	Positive	1	5	2	

*FIGO* International Federation of Gynecology and Obstetrics, *LVSI* lymphovascular space invasion, *y.o.* years old. * indicating *P* < 0.05, and ** indicating *P* < 0.01.

### Gal3 levels vary across a cohort of uterine cancer cell lines

Gal3 expression in uterine cancer cell lines was assessed by immunoblotting (Supplementary Fig. S2A). The levels of Gal3 expression observed in established USC cell lines (ARK1, ARK2, and SPEC2) were relatively greater than endometrioid endometrial AN3CA, HEC1A, and HEC1B cell lines. The Ishikawa cell line was the outlier; while it originated from a well-differentiated endometrioid carcinoma, it had moderate levels of Gal3 compared to the others. However, we find it to be a relatively aggressive line in our xenograft models (data not shown). Given our focus, we used the ARK1 and ARK2 cell lines for subsequent experiments. Gal3 can be differentially located within the cell [11], therefore ICC was performed using Anti-Gal3 antibody in the ARK1 and ARK2 cells. Gal3 was found to be widely distributed in the cytoplasm and nucleus in both cell lines (Supplementary Fig. S2B).

### The loss of Gal3 impacts cell proliferation and viability

**Fig. 2:**
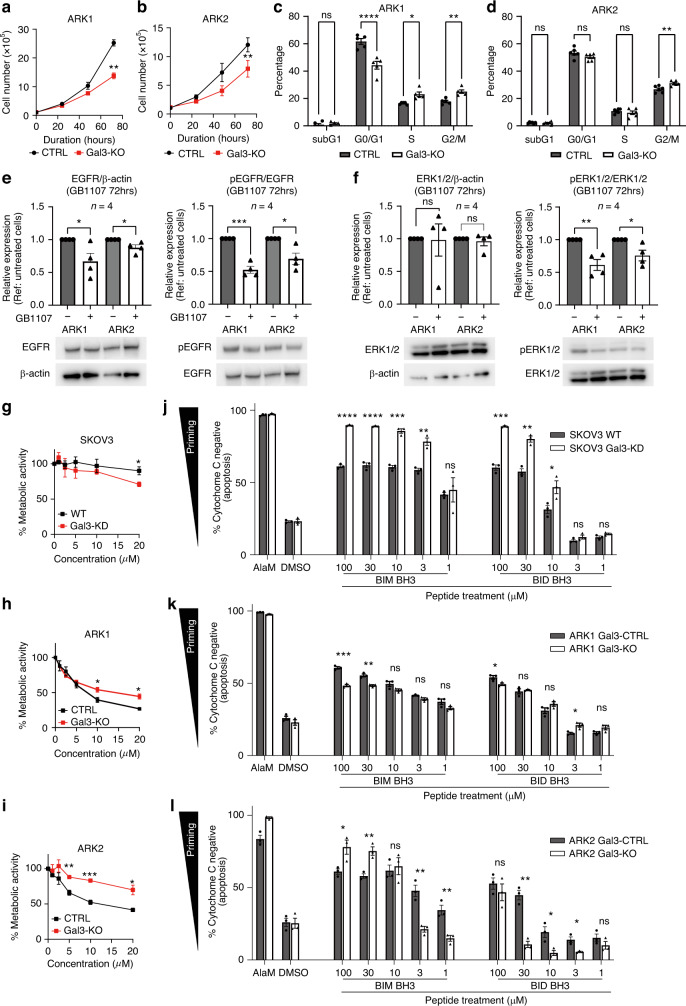
Loss of Gal3 effect on cell proliferation and cell viability. **a** cell counts in ARK1 cells, **b** cell counts in ARK2 cells, **c** The percentage of each stage of cell cycle in ARK1 cells. **d** The percentage of each stage of cell cycle in ARK2 cells. **e** Immunoblotting of cell cycle regulatory proteins representative of the G2/M phase in ARK1 cells. *E* Immunoblot of EGFR and pEGFR in lysates from ARK cell lines 72-hours post-treatment with GB1107. **f** Immunoblot of ERK1/2 and pERK1/2 in lysates from ARK cell lines 72-hours post-treatment with GB1107. **g** and **h** MTT assay using carboplatin in SKOV3 or ARK1 cells after 72 h exposure, respectively. **i** MTT assay using carboplatin in ARK2 cells after 72 h exposure, **j**, **k** and **L** BH3 profiling in SKOV3, ARK1 and ARK2 cells, respectively. In all experiments, Gal3 knockdown or knockout cells were compared to corresponding controls. Significance was calculated using *t*-test with * indicating *P* < 0.05, ** indicating *P* < 0.01, *** indicating *P* < 0.001, and **** indicating *P* < 0.0001. Significance was calculated using Šidák correction (cell cycle assay) and *t*-test (others) with * indicating *P* < 0.05, ** indicating *P* < 0.01,*** indicating *P* < 0.001 and **** indicating *P* < 0.0001.

ARK1 and ARK2 Gal3-KO cells were generated using the CRISPR/Cas9 system. Gal3 gene-editing was confirmed by immunoblotting and Sanger sequencing (Supplementary Fig. S2c, d). Cell counts were determined after 0, 24, 48, and 72 h to compare the ARK1 and ARK2 Gal3-CTRL and Gal3-KO lines. The number of viable cells after 72 h was higher in the Gal3-CTRL lines when compared to the Gal3-KO lines (ARK1: *p* = 0.0016, ARK2: *p* = 0.0063) (Fig. 2a, b). These results suggest that under baseline conditions, the loss of Gal3 impacted total cell number. We then assessed the baseline percentage of apoptotic/necrotic cells by flow cytometry. No difference in the percentage of early-phase apoptotic cells and late-phase apoptotic/necrotic cell in any cell lines was observed at baseline (Supplementary Fig. S3a–d). Collectively, these results imply that total loss of Gal3 influences proliferation rate and not cell death.

Subsequently, a cell cycle analysis was initiated with the same Gal3-CTRL and KO cells. A modest but significant increase in the percentage of cells in the G2/M phase was observed in the KO isotypes of both ARK1 and ARK2 cell lines compared to their relative controls (ARK1: *p* = 0.0017, ARK2: *p* = 0.0059). ARK1 cells showed a decrease in the G0/G1 phase (*p* = 0.0011) and an increase in the S phase (*p* = 0.004) (Fig. 2c, d). To further appreciate the change, the relative levels of key cell cycle regulatory proteins associated with the G2/M phase were evaluated by immunoblotting (Supplementary Fig. 3e, f). The expression level of cyclin A2 was increased in both Gal3-KO lines when compared to their counterparts (ARK1: *p* = 0.0393, ARK2: 0.015). Together, these results suggest that Gal3 contributes to USC proliferation, albeit more likely an indirect effect.

Gal3 can mediate a multitude of signaling pathways in different tumor cells in different contexts [26, 34–37]. Based on our previous work in ovarian cancer we suspected EGFR signaling may differ in Gal3-KO cells compared to their controls. At baseline, the EGFR and phosphorylated EGFR (pEGFR) levels were reduced in the ARK2 Gal3-KO cells compared to Gal3-CTRL cells (Supplementary Fig. S4a, b). These findings led us to assess the response to the Gal3-SMI, GB1107. ARK2 cells treated with GB1107 exhibited lower expression of EGFR at 12-hour post treatment (ARK1: *p* = 0.337, ARK2: *p* = 0.0074) compared to vehicle-treated cells. However, we observed a reduced level of pEGFR for both ARK1 and 2 cells post-treatment with GB1107 (ARK1: *p* = 0.0349, ARK2: *p* = 0.0014) (Supplementary Fig. 4c). At 72-hour post-treatment with GB1107 we observed a reduction in non-phosphorylated EGFR (ARK1: *p* = 0.0355, ARK2: *p* = 0.0258) and pEGFR compared to vehicle treated cells (ARK1: *p* = 0.0001, ARK2: *p* = 0.0122) (Fig. 2e). These results suggest Gal3 might influence total EGFR expression and the differences observed in the ratio of pEGFR/EGFR implies it may also impact EGFR phosphorylation. Additionally, ERK1/2, a downstream mediator of EGFR signaling was examined at 12- and 72-hour post-treatment with GB1107. Non-phosphorylated ERK1/2 levels remained unaffected by treatment with GB1107, but phosphorylated ERK1/2 (pERK1/2) levels were reduced at 12- (Supplementary Fig. 4d) and 72-hour (Fig. 2f) (at 12-hour ARK1: *p* = 0.0287, ARK2: 0.0352, at 72-hour: ARK1: p = 0.036, ARK2: 0.0246). We also assessed AKT signaling in Gal3-KO and controls as well as in response to GB1107. As expected, we observed no differences given both lines are known to be HER2 amplified and ARK1 having an activating PIK3CA mutation [19] (data not shown). The regulation of receptor stability and signaling through glycosylation reportedly [38] suggests that the glycosylation effects on receptors are dynamic, and small-magnitude changes may have potent downstream effects over time. Cell culture in a serum-containing media exposes the target cells to both growth factors and unknown amounts of galectins. Therefore, to further investigate how Gal3 may be mediating EGFR and ERK1/2 signaling in USC, ARK1 and ARK2 cells were cultured in serum-starved media for 6 h. Then, they were treated with 20 ng/ml of EGF + vehicle (DMSO) or 10 µM of GB1107. After 30 min, cells were harvested in lysate buffer and immunoblotting was performed for EGFR/pEGFR/ERK1/2/pERK1/2. Treatment with EGF and GB1107 did not change baseline levels of EGFR and ERK1/2 when compared with cells treated with EGF + vehicle (Supplementary Fig. S5a). However, the ratio of pEGFR to EGFR in response to EGF + GB1107 treatment group was less than those treated with EGF + vehicle in both lines (ARK1: *p* = 0.0436, ARK2: 0.0438; Supplementary Fig. S5b). Overall, these results indicate that loss or pharmacologic inhibition of Gal3 negatively impacts EGF-EGFR induced ERK phosphorylation and by default EGFR ERK signaling. The exact mechanisms by which Gal3 mediates EGF-EGFR-ERK1/2 signaling remain unclear given the potential for the small molecule inhibitor to bind and inhibit Gal3 interactions with a number of intracellular and extracellular proteins.

Previous work has shown that Gal3 can migrate from the nucleus to the cytoplasm, localizing near the mitochondria, where it can bind to proapoptotic BCL-2 family members [39] to promote drug resistance [40]. Consistent with this potential role in chemoresistance, we previously found knock-down of Gal3 in SKOV3 ovarian cancer cells rendered them more sensitive to carboplatin (Fig. 2g). We therefore tested whether the same was true for the ARK1 and ARK2 USC cell lines. In contrast to our previous observations in the ovarian cancer cell line, we did not see an increase in drug sensitivity in the Gal3-KO cells following treatment with carboplatin when compared to Gal3-CTRL cells and instead detected drug resistance (Fig. 2h, i). This led us to speculate that Gal3 may regulate apoptosis differently in USC cells as compared with ovarian cancer [28]. We tested this using BH3 profiling, which measures mitochondrial sensitivity to titrated doses of pro-apoptotic BH3 peptides by detecting cytochrome c release [28]. Cytochrome c release is the initiating step in mitochondrial apoptosis and cells that undergo this release in response to low doses of BH3 peptides are considered to be primed for apoptosis. Apoptotic priming has been previously shown to be a major contributor to cellular sensitivity or resistance to cancer therapeutics [41]. BH3 profiling revealed that SKOV3 Gal3-KD cells were more primed for apoptosis than the SKOV3 WT cells, as indicated by increased release of cytochrome c in response to both BIM and BID BH3 peptides (Fig. 2j–l). Both of these peptides can inhibit all pro-survival BCL-2 family proteins and also activate BAX and BAK [28], indicating that Gal3 expression is associated with an overall decrease in apoptotic priming.

BH3 profiling also showed ARK1 and ARK2 cells were relatively unprimed given the limited levels of cytochrome c release in response to even very high levels of pro-apoptotic BIM and BID BH3 peptides (10, 30 and 100 μM concentrations), which typically induce full release of cytochrome c in primed cancer cells [42]. Further, the differences between the ARK1 and ARK2 Gal3-KO cells varied across the doses of peptides used. For instance, the ARK1 Gal3-KO cells were slightly less sensitive to high doses of BIM BH3 peptides but slightly more sensitive to the low doses of BID while the opposite was evident in the ARK2 cells. Overall, this suggests Gal3 does not have a consistent effect on priming in the USC cells as it does in the ovarian cancer cells.

### Gal3 loss has a marked effect on stemness features

**Fig. 3:**
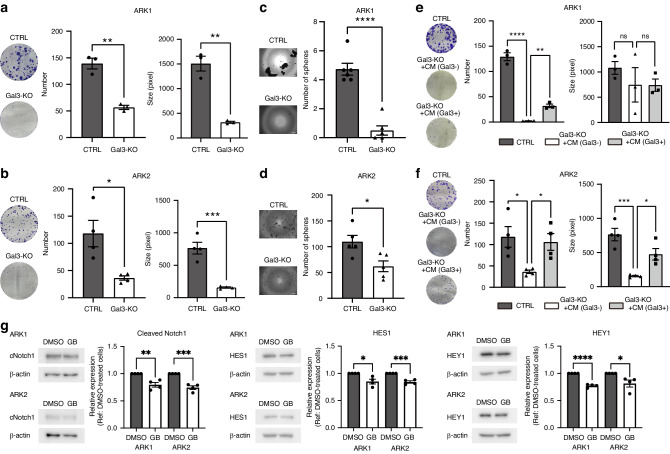
Loss of Gal3 impact on stem like properties. **a** Colony forming assay in ARK1 Gal3-CTRL and Gal3-KO cells. **b** Colony forming assay in ARK2 Gal3-CTRL and Gal3-KO cells. **c** Sphere forming assay in ARK1 Gal3-CTRL and Gal3-KO cells. **D** Sphere forming assay in ARK2 Gal3-CTRL and Gal3-KO cells. **e**, **f** Colony forming assay in ARK1 or ARK2 using CM from ARK2 Gal3-CTRL or ARK2 Gal3-KO CM from ARK2 Gal3-CTRL or ARK2 Gal3-KO, respectively. **g** Immunoblotting of cleaved Notch1, HES1, and HEY1. Significance was calculated by Dunnett’s test for the colony-forming assay using CM, while for other comparisons, the T-test was employed with * indicating *P* < 0.05, ** indicating *P* < 0.01, *** indicating *P* < 0.001, and **** indicating *P* < 0.0001.

Colony forming and sphere-forming assays were performed to further appreciate the impact of reduced or loss of Gal3. The Gal3-KO cell lines formed fewer and smaller colonies (number: ARK1: *p* = 0.0016, ARK2: *p* = 0.0159, size: ARK1: *p* = 0.0014, ARK2: *p* = 0.0006) (Fig. 3a, b). In addition, the number of spheres was decreased in Gal3-KO when compared to the Gal3-CTRL cells (ARK1: *p* < 0.0001, ARK2: *p* = 0.0176), suggesting the stem cell population may be reduced (Fig. 3c, d). To confirm whether these changes were due to Gal3, CM obtained from ARK2 Gal3-CTRL (Gal3 positive) and ARK2 Gal3-KO (Gal3 negative) cells were used for add-back experiments, which were performed using a colony-forming assay. A Gal3 ELISA assay revealed 1 ng/ml of Gal3 was evident in the CM obtained from ARK2 Gal3-CTRL cells. Independently, the extracellular vesicles (EVs) extracted from the CM from ARK2 Gal3-CTRL were also found to contain Gal3 (Supplementary Fig. S6). Independently, the addition of CM from ARK2 Gal3-CTRL to Gal3-KO cells resulted in a partial recovery of colony number (ARK1: *p* = 0.0096, ARK2: *p* = 0.04) and/or size (ARK1: *p* = 0.9996, ARK2: 0.0172) compared to the Gal3-KO cells treated with CM from ARK2 Gal3-KO cells (Fig. 3e, f). However, the lack of similar recovery with the addition of only recombinant human galectin 3 (rhGal3) (data not shown). To extend our investigations, GB1107 or vehicle (DMSO) was added with CM from ARK2 Gal3-CTRL cells to assess whether we would see a reduction in colony forming abilitysimilar to what we observed in the ARK-KO lines. The results showed a similar trend in that the number of colonies formed in the CM from ARK2 Gal3-CTRL in the presence of GB1107 were fewer in both cell lines and smaller in ARK2 Gal3-KO cell than those in the vehicle-control group (number: ARK1: *p* = 0.0022, ARK2: *p* = 0.0062, size: ARK1: *p* = 0.2439, ARK2: *p* = 0.0437) (Supplementary Fig. S7a, b). These results provide additional evidence Gal3 positively influences colony forming ability. However, while knockout and pharmacologic inhibition of Gal3 hampers colony-forming ability, rhGal3 had no significant impact on rescuing the reduced colony-forming ability observed on Gal3-KO cells. We postulate this lack of effect may be due, at least in part, to the structural differences between the recombinant and native protein. More specifically, native Gal3 can be found in monomeric or multimeric states within the nucleus, cytoplasm, and extracellular space [43]. Whether the recombinant human Gal3 protein used in this specific experiment can mimic all these various states is not fully appreciated. Moreover, the fact that Gal3 can bind to so many proteins, it is possible the rhGal3 added to the cultures was rapidly bound to other proteins in the media. Alternatively, rhGal3 could be readily cleaved and or degraded [43]. Despite the limited activity of rhGal3 observed, colony-forming activity was reduced by both knock-out and pharmacologic inhibition of Gal3 supporting its perceived role in contributing to stem-like properties.

To show additional evidence Gal3 supports stem like properties, we evaluated the relative levels of CD44, CD133, and CD117 positive populations and the percentage of cells displaying aldehyde dehydrogenases (ALDH) activity, in the ARK1 and ARK2 cell lines which have been shown to be enriched for cancer stem cells (CSCs) in other gynecologic cancers [44, 45] (Supplementary Fig. S8a–h). Flow cytometry revealed that CD44 and CD133 were inadequate for sorting CSCs in some lines given their already high baseline levels. However, the percentage of CD117 positive cells decreased in Gal3-KO of both lines (ARK1: *p* = 0.0149, ARK2: *p* = 0.0334). Similarly, the percentage of ALDH-active cells was reduced in both Gal3-KO lines compared to their controls (ARK1: *p* = 0.0446, ARK2: *p* = 0.0054). These findings along reduced sphere and colony forming ability imply loss of Gal3 negatively impacts stem like properties.

Gal3 has been reported to support stemness in ovarian cancer by activating the Notch1 signaling pathway [35–37], therefore we assessed the levels of intact and cleaved Notch1 (Notch1 intracellular domain (NICD)) in the ARK1 and 2 Gal3-CTRL and KO cells by immunoblotting. The levels of both Notch1 and cleaved Notch1 were less in the ARK1 and 2 Gal3-KO cells compared to their respective controls (Notch1: ARK1: *p* = 0.0038, ARK2: 0.0233, cleaved Notch1: ARK1: *p* = 0.0007, ARK2: *p* = 0.0366) (Supplementary Fig. S9). To investigate the effect of pharmacologic inhibition on Notch1 and downstream proteins, the levels of Notch1, HES1 and HEY1 were assessed in ARK1 and ARK2 cells in a sphere model. Briefly, ARK1 and ARK2 spheres were generated and cultured in media containing vehicle or 10 µM of GB1107 for 1 week, changing media containing vehicle or GB1107 every other day. Immunoblotting revealed that there was no difference in whole Notch1 levels between vehicle and GB1107-treated spheres, yet levels of cleaved Notch1 (ARK1: *p* = 0.003, ARK2: *p* = 0.0003), HES1(ARK1: *p* = 0.0133, ARK2: 0.0003), and HEY1 (ARK1: *p* < 0.0001, ARK2: 0.0257) were less in the GB1107-treated group compared to the vehicle-treated group (Fig. 3g). These results support the concept Gal3 is contributing to stem-like properties via Notch1 signaling. Independently, we investigated whether there were any differences in β-catenin expression given its reported role in stemness [45]. IHC and ICC analysis of the Gal3-CTLR and KO xenografts and cells revealed no significant difference between the two lines (data not shown). These findings imply that Gal3 might rely on Notch1 as well as other pro-stem signaling pathways to support stemness in USC.

### Effect of Gal3 on metastatic potential

**Fig. 4:**
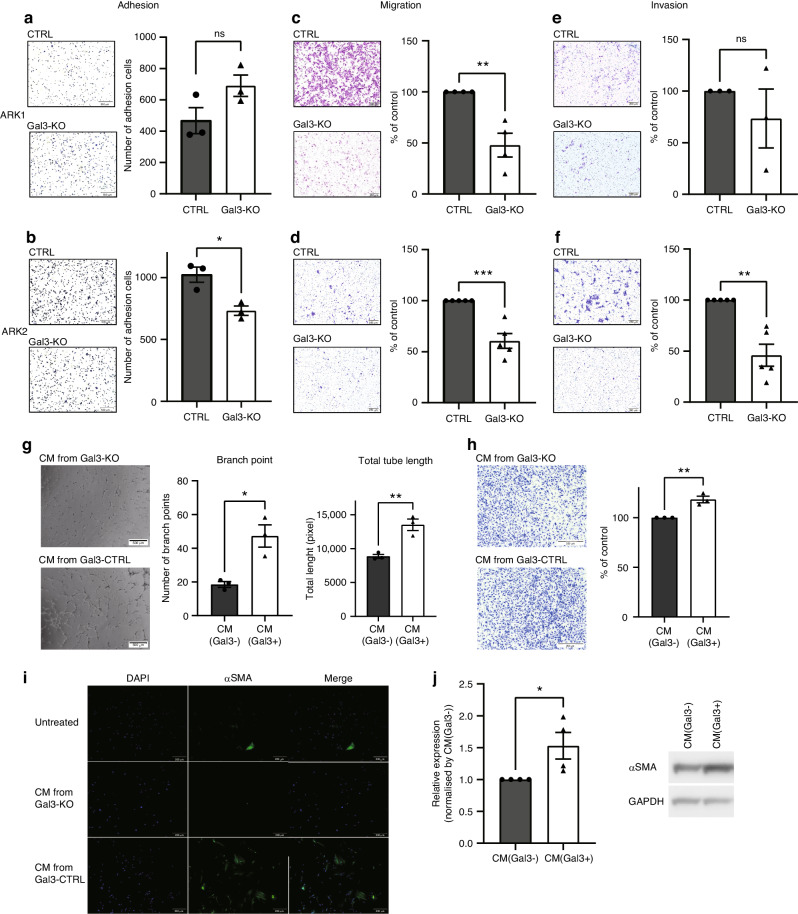
Loss of Gal3 effect on adhesion, migration, invasion potential and TME. **a** Fibronectin adhesion assay in ARK1 Gal3-CTRL and Gal3-KO cells. **b** Fibronectin adhesion assay in ARK2 Gal3-CTRL and Gal3-KO cells. **c** Trans-well assay in ARK1 Gal3-CTRL and Gal3-KO cells. **d** Trans-well assay in ARK2 Gal3-CTRL and Gal3-KO cells. **e** Matrigel invasion assay in ARK1 Gal3-CTRL and Gal3-KO cells. **f** Matrigel invasion assay in ARK2 Gal3-CTRL and Gal3-KO cells. **g** Tube-forming assay using HUVECs. **h** Angiogenesis invasion assay using HUVECs. **I** ICC images fibroblasts to detect differences in αSMA (100× magnification). J) Immunoblotting for αSMA and densitometric quantified graph. Significance was calculated using t-test with * indicating *P* < 0.05, ** indicating *P* < 0.01, and *** indicating *P* < 0.001.

The role of Gal3 in tumor cell adhesion, invasion, migration, and metastatic potential in different solid tumor types varies widely and often has opposing actions [46, 47]. Initially, we examined the effect of Gal3 loss on adhesion, migration, and invasion properties in the ARK cells. An adhesion assay using a fibronectin-coated plate was performed to examine base line cell adhesion rates in the ARK1 and 2 Gal3-CTRL and Gal3-KO lines (Fig. 4a, b). Though ARK1 cells had no difference (*p* = 0.1064), the number of ARK2 Gal3-KO cells that adhered was less compared to ARK2 Gal3-CTRL cells (*p* = 0.0158) implying there is likely some patient-to-patient diversity.

To explore this further, a trans-well assay was performed to assess the impact of loss of Gal3 on cell migration. The percentage of cells migrating to toward the FBS was different among the Gal3-CTRL and KO cells (ARK1: *p* = 0.0042, ARK2: *p* = 0.0006) (Fig. 4c, d). Only the ARK2 cells showed the same trend in response to loss of Gal3 in the Matrigel invasion assay whereas ARK1 cells did not differ (ARK1: *p* = 0.4031, ARK2: *p* = 0.001) (Fig. 4e, f).

Also, to confirm the effects of pharmacological Gal3 inhibition, ARK1 and ARK2 cells were cultured in complete media with DMSO (vehicle) or 10 µM of GB1107 for 72 h and then submitted to the fibronectin adhesion, transwell migration, and invasion assay as described above. The effects observed in response to the Gal3-SMI were similar to what we observed in the Gal3-KO cells. Specifically, we observed a decrease the number of adhered cells in ARK2 (ARK1: *p* = 0.8178, ARK2: *p* = 0.0389), a decrease number of migrating cells (ARK1: *p* < 0.0001, ARK2: *p* = 0.0001), and a decreasing number of invading cells (ARK1: *p* = 0.014, ARK2: *p* < 0.0001) compared to DMSO treated cells (Supplementary Fig. S10a–f).

To investigate the effect of Gal3 loss on angiogenesis, the tube-forming assay and angiogenesis invasion assay were performed using HUVECs. The total tube length and number of branch points were measured as outcomes. The well coated with the conditioned media (CM) harvested from ARK2 Gal3-CTRL cells had more branch points (*p* = 0.0139) and a longer total tube length (*p* = 0.0064) (Fig. 4g). The tube-forming assay was repeated using rhGal3 combined with CM from Gal3-KO cells to clarify the Gal3 effect. The tube length and the number of branch points were longer and larger on rhGal3 and CM from ARK2 Gal3-KO cells coated wells, (branch point: *p* = 0.0367, length: *p* = 0.0205) (Supplementary Fig. S11a).

For the angiogenesis invasion assay, the Matrigel-coated insert was primed with CM from ARK2 Gal3-CTRL or CM from ARK2 Gal3-KO cells. Twenty-four hours post-seeding of HUVECs cells, more cells infiltrated the insert primed with CM from Gal3-CTRL cells than those primed with CM from KO cells (*p* = 0.0054) (Fig. 4h). Independently, the Matrigel-coated insert was primed with CM from ARK2 Gal3-KO cells and 5 µg/mL of rhGal3 or vehicle in the angiogenesis invasion assay. Priming with rhGal3 increased the number of infiltrating cells compared to CM from Gal3-KO cells plus vehicle treatment (*p* < 0.0001) (Supplementary Fig. S11b). These findings suggest Gal3 may serve as a potential direct inducer of angiogenesis.

To further support the idea Gal3 can positively influence angiogenesis, cultured HUVECs were exposed to CM from ARK2 Gal3-CTRL containing vehicle or GB1107 at 10 µM to determine if like the knockout model, pharmacological inhibition of Gal3 negatively impacted tube forming ability. Reduced total tube length (*p* = 0.0064) and fewer branch points (*p* = 0.0132) were observed in the HUVECs cultured with CM from ARK2 Gal3-CTRL cells with GB1107 compared with HUVECs cultured with vehicle (Supplementary Fig. S11c).

To begin to assess the impact of Gal3 on other aspects of the tumor microenvironment (TME), we evaluated whether the presence or absence of Gal3 would influence the transition of normal fibroblasts towards a CAF-like phenotype using αSMA as an endpoint marker. The expression of αSMA in IMR90 cells exposed to CM from ARK2 Gal3-CTRL cells was notably stronger than fibroblasts exposed to CM from ARK2 Gal3-KO cells, as indicated by ICC (Fig. 4i, j). Others have shown TGF-β to induce the transition of normal fibroblasts towards a CAF-like phenotype, as determined by an increase in αSMA [48, 49]. Thus, we used this strategy as a positive control. Exposure of fibroblasts to 10 ng/ml of TGF-β did increase αSMA compared with PBS (vehicle). Interestingly, we also observed a decline in the levels of Gal3 in the fibroblasts exposed to TGF-β when compared to vehicle exposure (Supplementary Fig. S12a). We then cultured fibroblasts in complete media with DMSO (vehicle of GB1107) or 10 µM of GB1107 for 48 h. αSMA expression levels were then evaluated. Adding GB1107 to complete media did not change the baseline expression level of αSMA of fibroblasts compared with DMSO control, suggesting endogenous Gal3 was not actively influencing a transition to a CAF phenotype. We then added DMSO (vehicle) or 10 µM of GB1107 with the Gal3-positive CM, thinking it would offset the increase in αSMA observed in response to exposure of the fibroblasts to Gal3-positive CM. However, there was no change in αSMA expression between CM + DMSO-treated fibroblasts and those treated with CM + GB1107 (Supplementary Fig. S12b). The lack of change in αSMA levels in response to the pharmacologic inhibition of Gal3 in this experiment led us to speculate that the increase in αSMA observed in the fibroblasts exposed to CM from ARK2 Gal3-CTRL cells compared with CM from ARK2 Gal3-KO cells was more likely an indirect compensatory effect resulting from total loss of Gal3 vs. acute inhibition.

### Confirmation of loss of Gal3 effect using in vivo models and patient-derived organoids

**Fig. 5:**
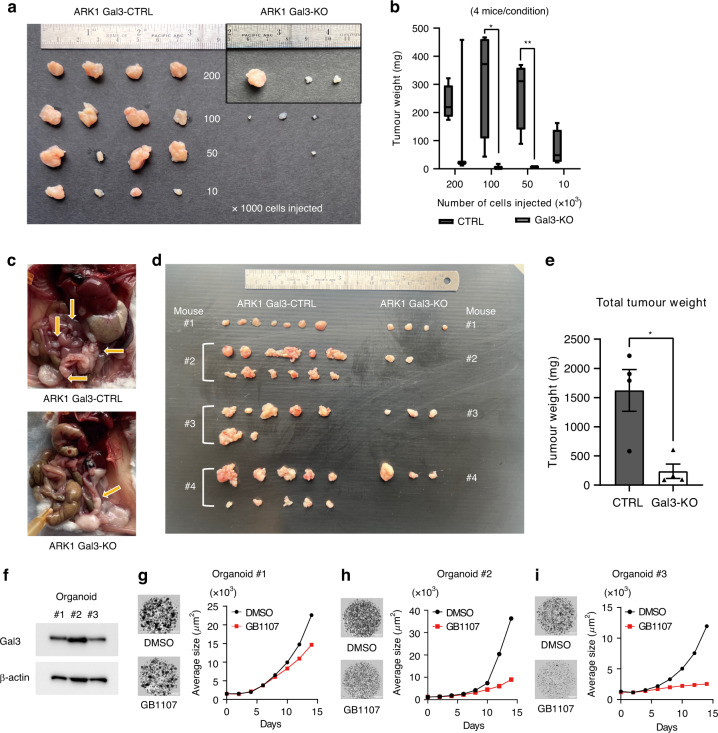
The impact of loss or inhibition of Gal3 in patient-derived organoids and xenograft models. **a** Gross image of tumors generated by injection of ARK1 Gal3-CTRL or Gal3-KO in the flanks. **b** Tumor weight in each arm. **c** Differences in tumor establishment and peritoneal spread following intraperitoneal injection of ARK1 Gal3-CTRL and Gal3-KO cells into immunocompromised mice. **d** Gross assessment of tumors in the IP mouse model. **e** Total tumor weight. **f** Gal3 levels in each PDO line. **g** **h**, **i** Image of organoids at day 14 and the growth curve depicting average size of organoid number 1, number 2, and number 3 after exposure to the vehicle/GB1107 (all *n* = 3 technical replicates). Significance was calculated using t-test with * indicating *P* < 0.05, and ** indicating *P* < 0.01.

To validate our in vitro findings, an in vivo serial dilution assay was performed to assess Gal3 loss on tumorigenesis. Gal3-CTRL cells formed tumors relatively quickly, however only one mouse injected with Gal3-KO cells had a palpable tumor. Moreover, the number of tumors formed from the Gal3-KO cells was markedly reduced compared to isotype controls (ARK1 Gal3-CTRL: 16/16, Gal3-KO: 7/16). Tumors generated in mice injected with ARK1 Gal3-CTRL cells were larger than those injected with the Gal3-KO cells (Injected with 200,000 cells: *p* = 0.6115, 100,000 cells: *p* = 0.0452, 50,000 cells: *p* = 0.0051, 10,000 cells: *p* = 0.0716) (Fig. 5a, b). Subsequent analysis of Ki67 by immunohistochemistry (IHC) showed no difference, but pHH3 staining visually showed a higher percentage of positive cells in Gal3-CTRL cells, suggesting while cells were entering the cell cycle at equal rates more Gal3-CTRL cells may have undergone mitosis than their counterparts (Supplementary Fig. S13).

The effect of loss of Gal3 on tumor cell implantation was confirmed using an IP model. One million cells of ARK1 Gal3-CTRL and KO cells were injected into NSG mice. Sixty days later, mice injected with Gal3-CTRL, bloody ascites was observed, and numerous disseminated lesions were found in the mesentery, retroperitoneum, and around the uterus and ovaries. In contrast, mice injected with Gal3-KO cells showed fewer lesions compared to the CTRL group. The total weight of IP lesions was higher in the Gal3-CTRL group (*p* = 0.0107) (Fig. 5c–e).

While knockout of Gal3 with CRSPR/Cas9 technology is an effective tool for better understanding its contribution to the pathobiology of uterine malignancies, there are pharmacologic inhibitors of Gal3 in Phase I trials, including the Gal3-SMIs TD139 and GB1107. Both were tested in our in vitro assays. While both were effective at high concentrations, GB1107 was more effective overall at the lower concentrations and had less off-target effects (as evidenced in the Gal3-KO cells) (Supplementary Fig. S14a–h). We subjected established PDOs to GB1107. The organoids were confirmed to be Gal3 positive by immunoblotting (Fig. 5f) and organoid growth was reduced after 14 days of exposure to GB1107 (Fig. 5g–i).

## Discussion

Gal3 has been implicated as a contributor to many of the aggressive features associated with basic tumor patho-biology including, but not limited to metastatic and invasive potential, immune suppression, and treatment resistance in some solid tumors, such as breast and prostate cancer [12, 40, 50]. Gal3 expression has been reported in the highly aggressive USC, although a thorough assessment of its expression in concordance with the clinicopathological features along with detailed functional studies designed to assess the role of Gal3 in promoting the pathology of the USC subtype was lacking. Our analysis of *LGALS3* expression in the uterine cancer in the TCGA revealed that elevated *LGALS3* expression was associated with worse clinical outcome in patients diagnosed with USC. We further demonstrate those patients with USC with no or a low level of Gal3 in their primary tumors were less likely to present with the more advanced stages, LVSI, and lymph node metastases. Moreover, our investigations using in vitro and in vivo pre-clinical models of USC illustrated for the first time that Gal3 has the potential to influence either directly, or indirectly cell proliferation, stemness, cell adhesion, migration, invasive potential, and or TME, supporting the concept that Gal3 likely contributes to the more aggressive clinical properties of USC.

The limited reports describing the expression of Gal3 in uterine tumor samples often differed in terms of their relative levels in diverse uterine histologies, grade and or stage evaluated. Some suggested high expression of Gal3 was associated with high-grade histology, but others concluded the opposite [17, 18]. This discrepancy was likely due to differing sample size, histologies, or antibodies etc. Moreover, some studies focused on Gal3 expression in only tumor cells of either the primary and or metastatic lesions, while others focused on the stroma expression [16, 18]. There has also been limited focus on the intracellular and extracellular distribution of Gal3 which given its reported pernicious nature could readily influence a multitude of different biologic aspects. We observed varied intra-cellular levels of Gal3 expression across all our different pre-clinical models, which could influence its role.

Our recent studies in preclinical breast and ovarian cancer models suggested inhibition of Gal3 could reduce metastatic tumor burden [15]. The correlation with *LGALS3* and Gal3 with poor outcome, along with the highly aggressive nature, metastatic potential, and likelihood of recurrence of USC led us to question whether Gal3 was a major contributor to its clinicopathological features and if targeting Gal3 might be of value to patients with USC. To this end, we needed to better understand the functional roles of Gal3 in USC.

There are various reports on the effect of Gal3 on proliferation in other solid tumor types, albeit again somewhat controversial [51–53]. Our study demonstrated that Gal3-KO USC lines proliferated at a slower rate than their respective controls, suggesting that Gal3 may only contribute modestly to base line cell proliferation. The mechanism(s) mediating this effect could be related to Gal3 influence on EGFR signaling as reported [26, 34], and cell cycle regulatory proteins such as Cyclin A2, which may cause G2/M arrest. However, considering the previous findings [51–53], Gal3’s impact on cell proliferation is likely to vary among tumor type, histology, and level of focal expression of Gal3, requiring further investigation.

Gal3 has been associated with apoptosis regulation and is involved in chemotherapy sensitivity [14]. However, the relationship between Gal3 and these factors in USC was opposite of what was expected, as chemoresistance was enhanced by the loss of Gal3. Since Gal3-KO decreased the cell proliferation rate, we considered the possibility of weakened effect of cytotoxic drugs which rely on rapid cell turnover. Further, the BH3 profiling results indicated that the effects of Gal3 on apoptosis sensitivity in USC varies among different lines and, given what we observed in our ovarian cancer model, could differ with tumor type, histology and molecular or oncogenic drivers for each patient. Further multimodal studies on the role of Gal3 in chemoresistance are required.

Given the aggressive nature, high incidence of resistance and recurrence of USC it was of particular interest to discern if Gal3 contributed to stemness features in this histophenotype. Gal3 is implicated in promoting stemness in other solid tumors [51, 54–58] but there are no known reports of Gal3 contributing to stemness in uterine cancers. How Gal3 impacts stemness varies among malignancies [54, 55, 58]. In our study, loss of Gal3 markedly decreased the percentage of CD117 positive cells and reduced ALDH activity which was concurrent with decreased ability to form colonies, spheres and tumorigenesis. Also, in line with what was observed in ovarian cancer [58], the expression of NICD was decreased in the USC cell line when Gal3 was knocked out or pharmacologically inhibited, suggesting that Gal3 may support stem-like properties through the same pathway. However, it is important to note that we cannot rule out the possibility that Gal3-Notch1 signaling can also influence non-stem cancer cells. While contributions of Notch1 in gynecologic cancer and cancer stem cells have been described previously [35–37], other stemness-associated pathways such as Wnt signaling could not be ruled out. However, our data, as well as a previous report suggesting [59] nuclear β-catenin expression was limited in a cohort of USC, hinting that the Gal3 impact on stemness we observed is not mediated via β-catenin in USC.

Investigations of other solid tumors [51, 52, 60] reported Gal3 participated in adhesion of cells to the extracellular matrix, and promoted migration, and invasion. In the present study, the loss of Gal3 negatively impacted cell adhesion, migration and or invasion in at least one of the genetically modified USC lines. Similar results were observed with pharmacologic inhibition albeit modestly.

The TME plays a major role in the progression of tumors. Similar to what others reported [13], Gal3 induced characteristics of angiogenesis as evidenced by tube-forming and invasion assays using HUVECs suggesting it can promote a favorable tumor growth environment. CAFs also support primary tumor growth, angiogenesis, metastasis, and resistance to therapy. We were not able to find previous publications describing whether Gal3 contributing to fibroblasts transition to CAFs. In the present study, we found that fibroblasts cultured in CM from Gal3-positive cells expressed more αSMA, a marker of CAFs, than cells cultured in CM from Gal3-KO. Like others we observed that treatment with TGF-β resulted in an increase in αSMA concurrent with a shift towards CAFs [48, 49]. We showed this increase in αSMA was inversely associated with a decrease in endogenous Gal3. Subsequent experiments with GB1107 exposure of fibroblasts or exposure of fibroblasts to CM from Gal3-positive cells with GB1107 failed to alter the levels of αSMA relative to their controls. We postulated this lack of effect by the pharmacologic inhibition of Gal3 compared to the differences observed in the knockout models was likely a compensatory or indirect effect elicited by the total loss of Gal3 as opposed to an acute effect of a Gal3 inhibition.

Overall, our in vivo experiments highlighted the cumulative effect of loss of Gal3 by showing the impact of loss of Gal3 on tumor formation and growth in the subcutaneous model, which could be due in part to Gal3 role in promoting stemness. The Gal3-KO cells also formed fewer and smaller lesions than Gal3-CTRL cells in our in vivo dissemination model. The addition of the Gal3 pharmacologic inhibitor reduced the growth of a cohort of patient-derived USC organoids relative to the vehicle-treated controls. All in all, our results suggest that Gal3 can contribute via a variety of mechanisms to the highly aggressive nature of USCs.

There are recognized limitations to this study. Albeit a relatively rare disease, the association between Gal3 expression and clinicopathological features in patient tissues revealed patients with low Gal3 expression were less likely to have metastatic disease, the clinical implication of this finding needs to be prospectively evaluated. After the initial evaluation of multiple endometrial cancer cell lines, two established USC cell lines were used to generate Gal3-KO lines, and cell function was examined. However, these outcomes only represent the impact of the complete knockout of Gal3. Given the potential context-specific differing biological roles of Gal3, disruption of specific Gal3 protein-protein interactions with antibodies or SMIs would also likely have differing outcomes. While the pre-clinical models provide additional confidence, experiments using optimized Gal3 inhibitors at lower nanomolar concentrations are required to provide confidence that targeting Gal3 might be a viable option as a single agent or in combination with cytotoxic are needed.

In conclusion, this study provides strong evidence that Gal3 can contribute to USC cell proliferation, stemness, migration, invasiveness, and a tumor-promoting microenvironment. Given its potential contribution varies among the different lines and models it was rare that Gal3 didn’t contribute to many of these features contributing to its aggressive nature and poor prognosis Consequently, strategies targeting Gal3-specific interactions should be expanded to include USC preclinical models to assess their efficacy in combatting this highly aggressive disease.

### Supplementary information

Supplementary data

ARRIVE Compliance Questionnaire

## Data Availability

The data generated in this study are available upon request from the corresponding author.
