# Supplementary material for: Targeting Galectin 3 illuminates its contributions to the pathology of uterine serous carcinoma
Source: Br J Cancer. 2024 Mar 4;130(9):1463–76. doi: 10.1038/s41416-024-02621-x (PMC11058234; doi:10.1038/s41416-024-02621-x)

Supplementary figures

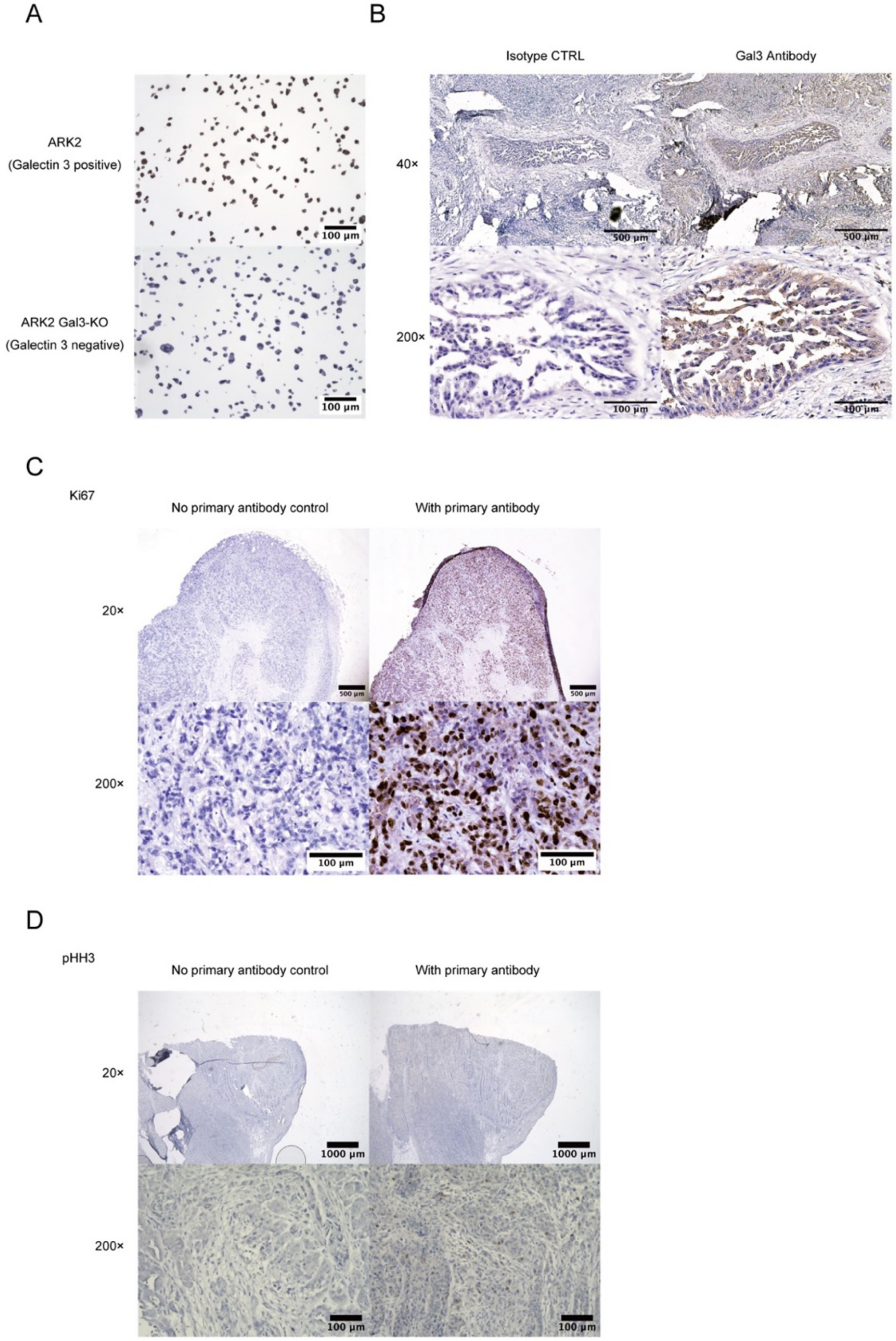

Supplementary Fig. S1: Validation of antibodies. A) Validation of anti-Gal3 antibody using Gal3 positive control (ARK2) and negative control (ARK2 Gal3-KO cells). B) Validation of anti-Gal3 antibody using isotype control. C) Validation of anti-Ki67 antibody. D) Validation of anti-pHH3 antibody.

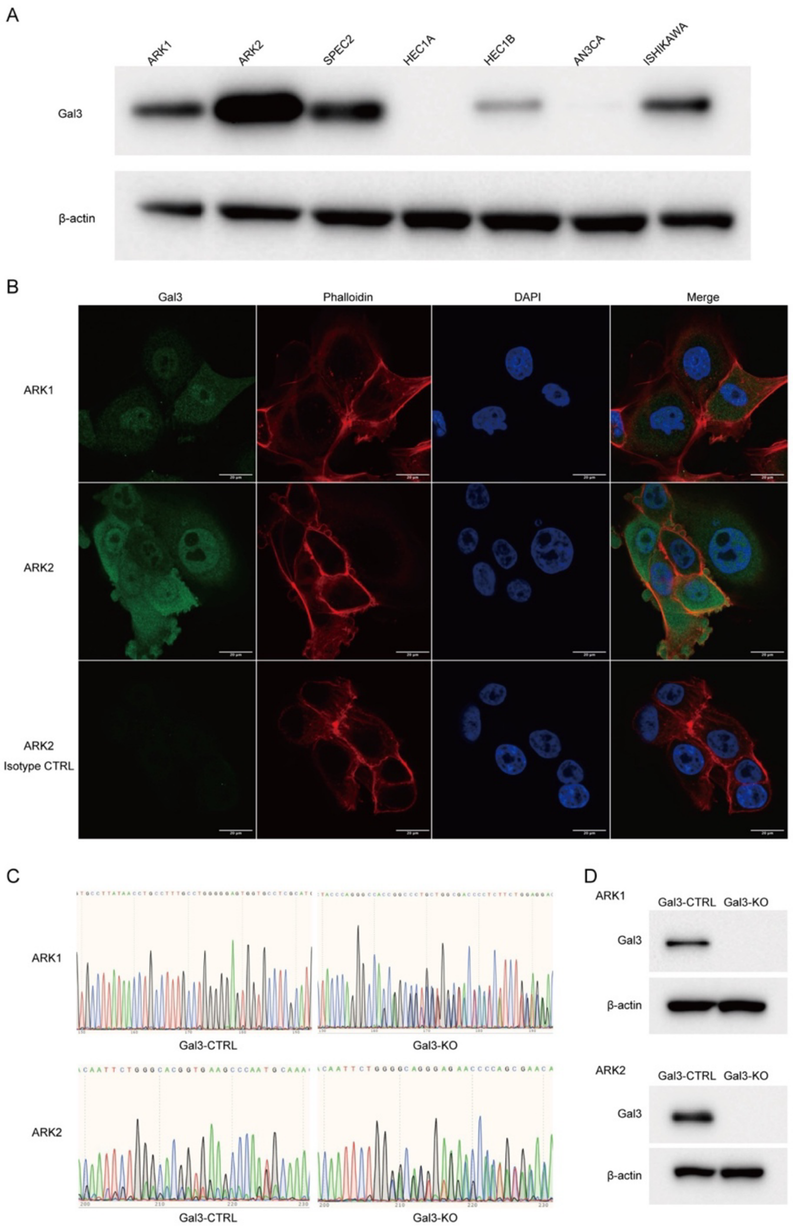

Supplementary Fig. S2: Confirmation of Gal3 expression, distribution, and Gal3-KO. A) Gal3 expression in endometrial cancer cell lines. ARK1, ARK2, and SPEC2 are derived from USC, while HEC1A, HEC1B, AN3CA, and Ishikawa cells were derived from endometrioid cancer. B) ICC images of Gal3 distribution. Distribution of Gal3 in ARK1 and ARK2 cells detected by immunofluorescence. In both cell lines, Gal3 was found to be distributed in both the cytoplasm and the nucleus. C) Sanger sequence of ARK1 and ARK2 Gal3-KO cells. D) Immunoblotting of ARK1, ARK2 Gal3-CTRL and Gal3-KO cells.

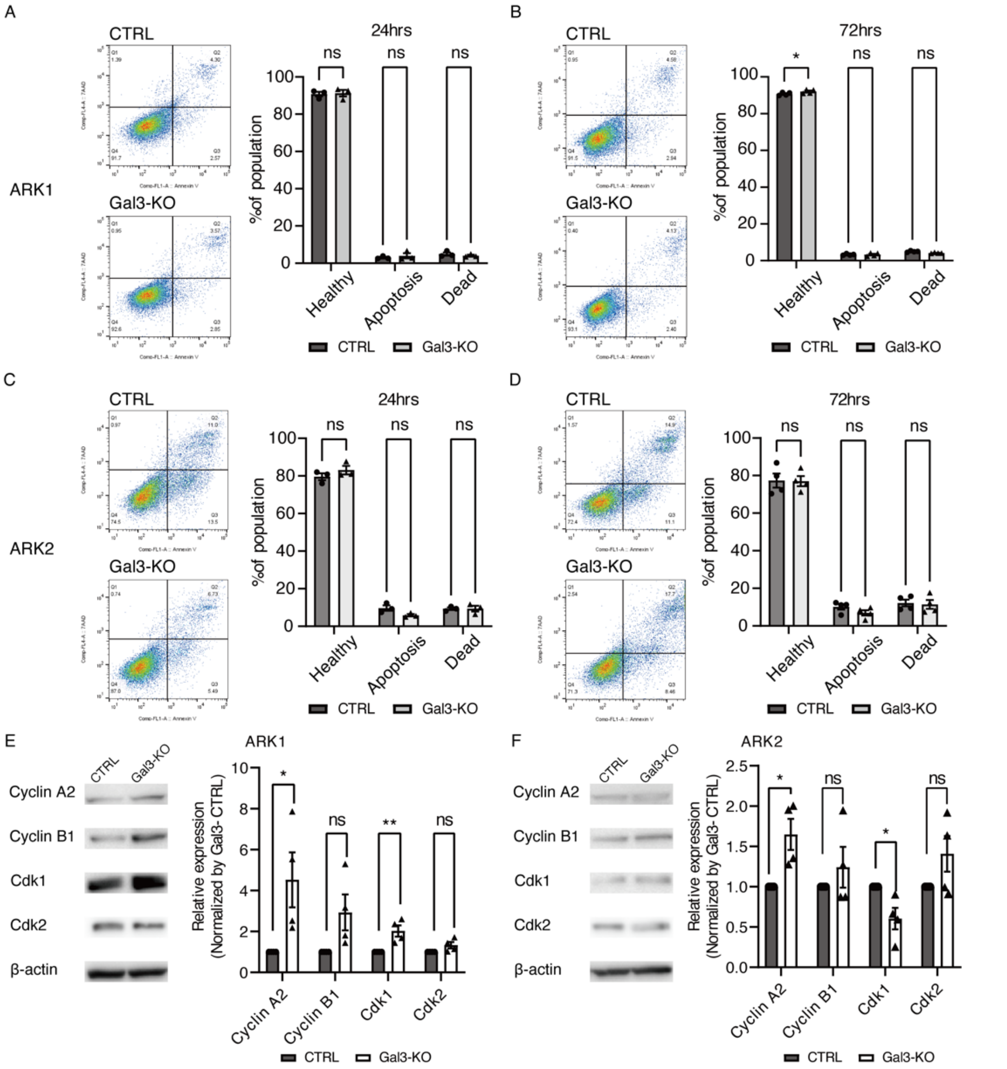

Supplementary Fig. S3: Effect of loss of Gal3 on cell viability baseline and cell cycle. A) Representative flow cytometry images and percentage of live/dead cells in ARK1 cells after 24 hours-incubation. B) Representative flow cytometry images and percentage of live/dead cells in ARK1 cells after 72 hours incubation. C) Representative flow cytometry images and percentage of live/dead cells in ARK2 cells after 24 hours-incubation. D) Representative flow cytometry images and percentage of live/dead cells in ARK2 cells after 72 hours incubation. E) Immunoblotting of cell cycle regulatory proteins representative of the G2/M phase in ARK1 cells. F) Immunoblotting results of cell cycle regulatory protein representative of the G2/M phase in ARK2 cells. Significance was calculated using t-test with * indicating $P < 0.05$, and ** indicating $P < 0.01$.

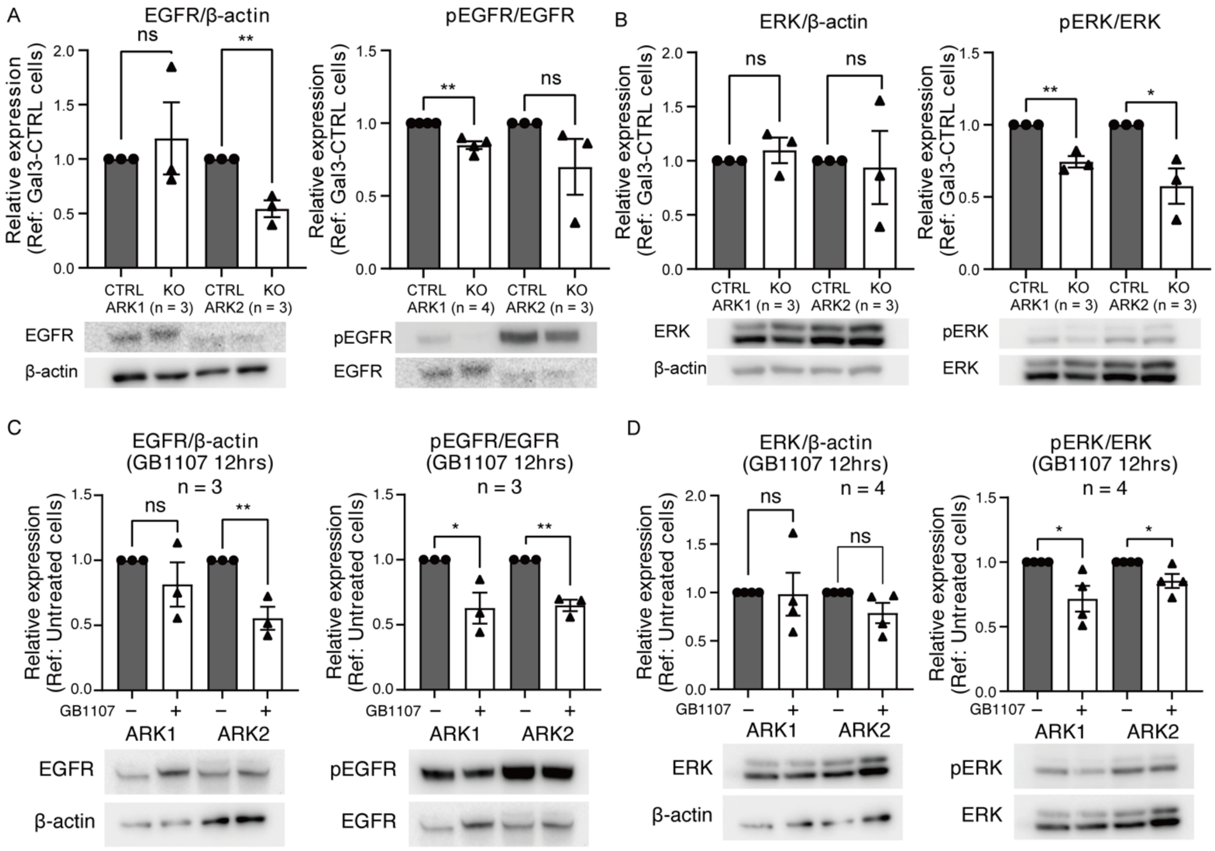

Supplementary Fig. S4: Baseline EGFR and ERK expression in Gal3-CTRL and Gal3-KO cells and acute effect of inhibition of Gal3 on EGFR and ERK expression. A) Immunoblot of EGFR and pEGFR. B) Immunoblot of ERK and pERK. C) Immunoblot of EGFR and pEGFR in lysates from ARK cell lines 12-hours post treatment with GB1107. D) Immunoblot of ERK and pERK in lysates from ARK cell lines 12-hours post treatment with GB1107. Significance was calculated using t-test with * indicating P < 0.05, and ** indicating P < 0.01.

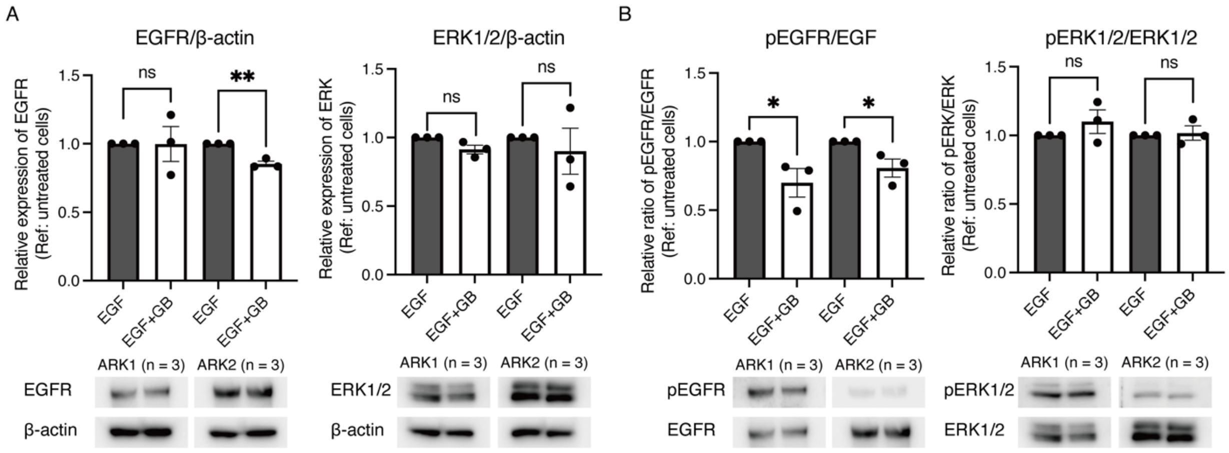

Supplementary Fig. S5: Acute effect of EGF + GB1107 administration on expression of EGFR and ERK and phosphorylation of EGFR and ERK1/2 in a serum free culture environment. A) Immunoblot of EGFR and ERK1/2 in ARK cell lines 30 minutes post treatment with EGF + vehicle or EGF + GB1107. B) Immunoblot of pEGFR and pERK in ARK cell lines 30 minutes post treatment with EGF + vehicle or EGF + GB1107. Significance was calculated using t-test with * indicating $P < 0.05$, and ** indicating $P < 0.01$.

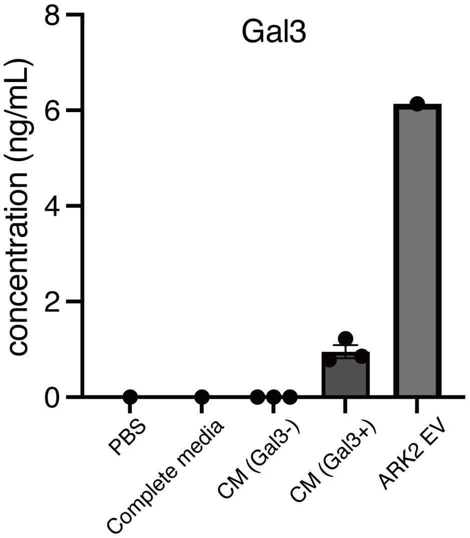

Supplementary Fig. S6: Gal3 concentration in conditioned media and extracellular vesicles isolated from ARK2 culture media as measured by ELISA.

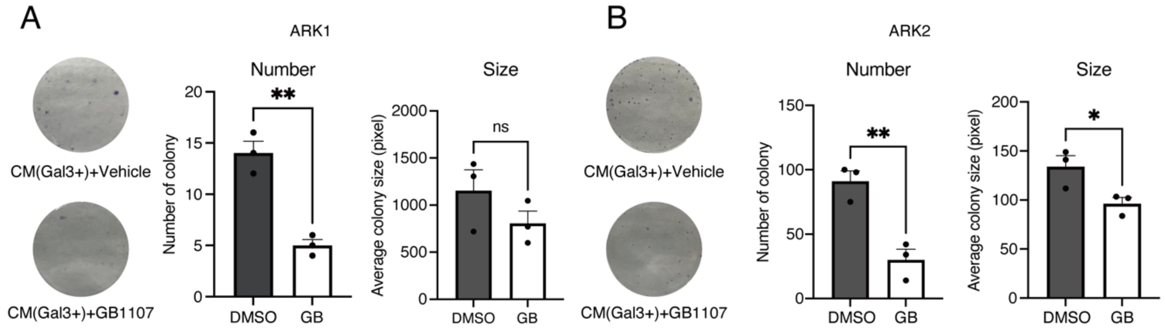

Supplementary Fig. S7: Determining if pharmacologic inhibition of Gal3 influences colony forming ability. A) Colony forming ability (Number and size of the colonies) of ARK1 Gal3-KO cells exposed to CM from ARK2 Gal3-CTRL + vehicle (DMSO) or GB1107 for 7 days. B) Colony forming ability (Number and size of the colonies) of ARK2 Gal3-KO cells exposed to CM from ARK2 Gal3-CTRL + vehicle (DMSO) or GB1107 for 7 days. t-test was employed with * indicating $P < 0.05$, and ** indicating $P < 0.01$.

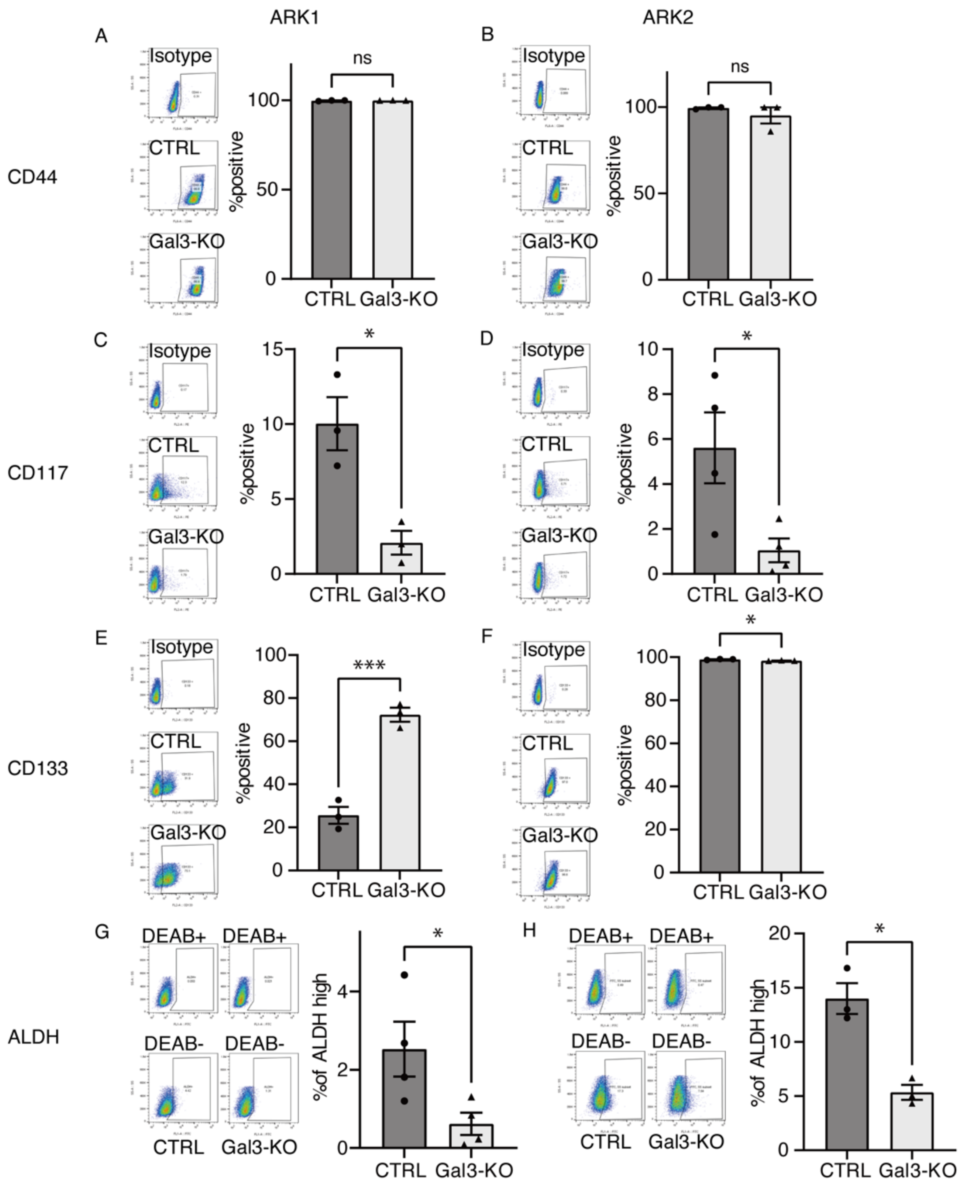

Supplementary Fig. S8: Expression of cancer stem markers. A) Percentage of CD44 positive cells in ARK1. B) Percentage of CD44 positive cells in ARK2. C) Percentage of CD117 positive cells in ARK1. D) Percentage of CD117 ARK2 positive cells. E) Percentage of CD133 positive cells in ARK1. F) Percentage of CD133 positive cells in ARK2. G) Percentage of ARK1 ALDH-activated cells. H) Percentage of ARK2 ALDH-activated cells. Significance was calculated using t-test with * indicating $P < 0.05$, and ** indicating $P < 0.01$.

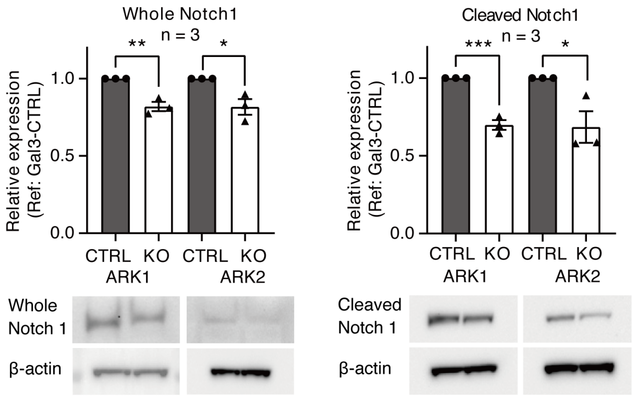

Supplementary Fig. S9: Immunoblotting of whole Notch 1 and cleaved Notch 1in ARK1 and ARK2 Gal3-CTRL and Gal3-KO cell lines. t-test was employed with * indicating P < 0.05, ** indicating P < 0.01, and *** indicating P < 0.001.

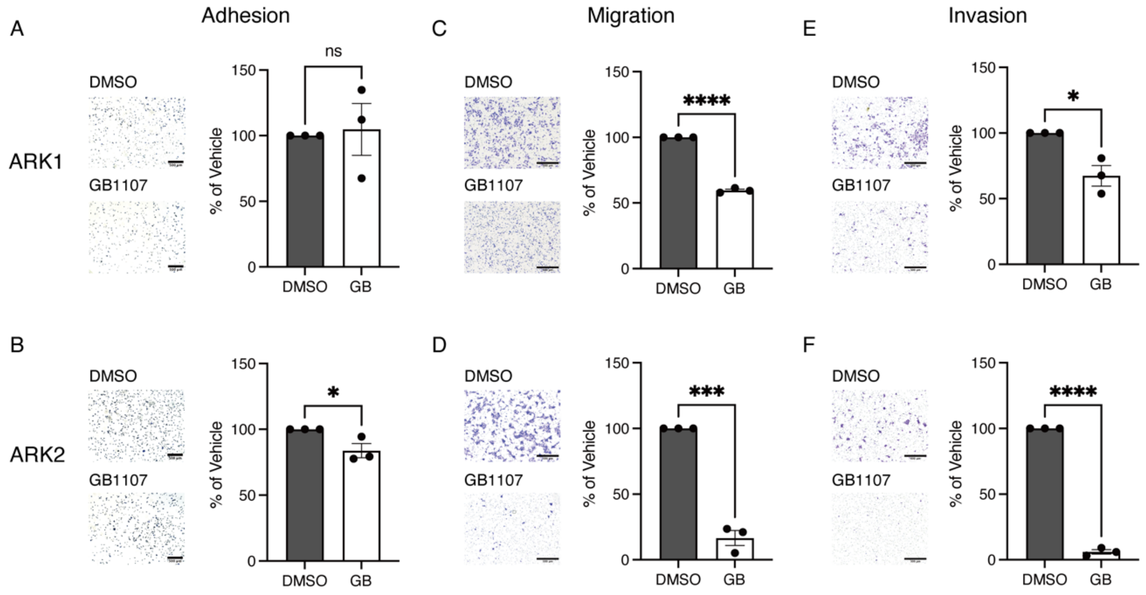

Supplementary Fig. S10: Impact of pharmacologic inhibition of Gal3 on adhesion, migration, and invasion potential. ARK1 and ARK2 cells were exposed to DMSO (vehicle) or 10 µM of GB1107. After 72 hours, cells were collected and submitted to: fibronectin adhesion assay in ARK1 (A) and ARK2 (B) cells, trans-well assay in ARK1 (C) and ARK2 (D) cells, and Matrigel invasion assay in ARK1 (E) and ARK2 (F) cells. Significance was calculated using t-test with * indicating P < 0.05, *** indicating P < 0.001, and **** indicating P < 0.0001

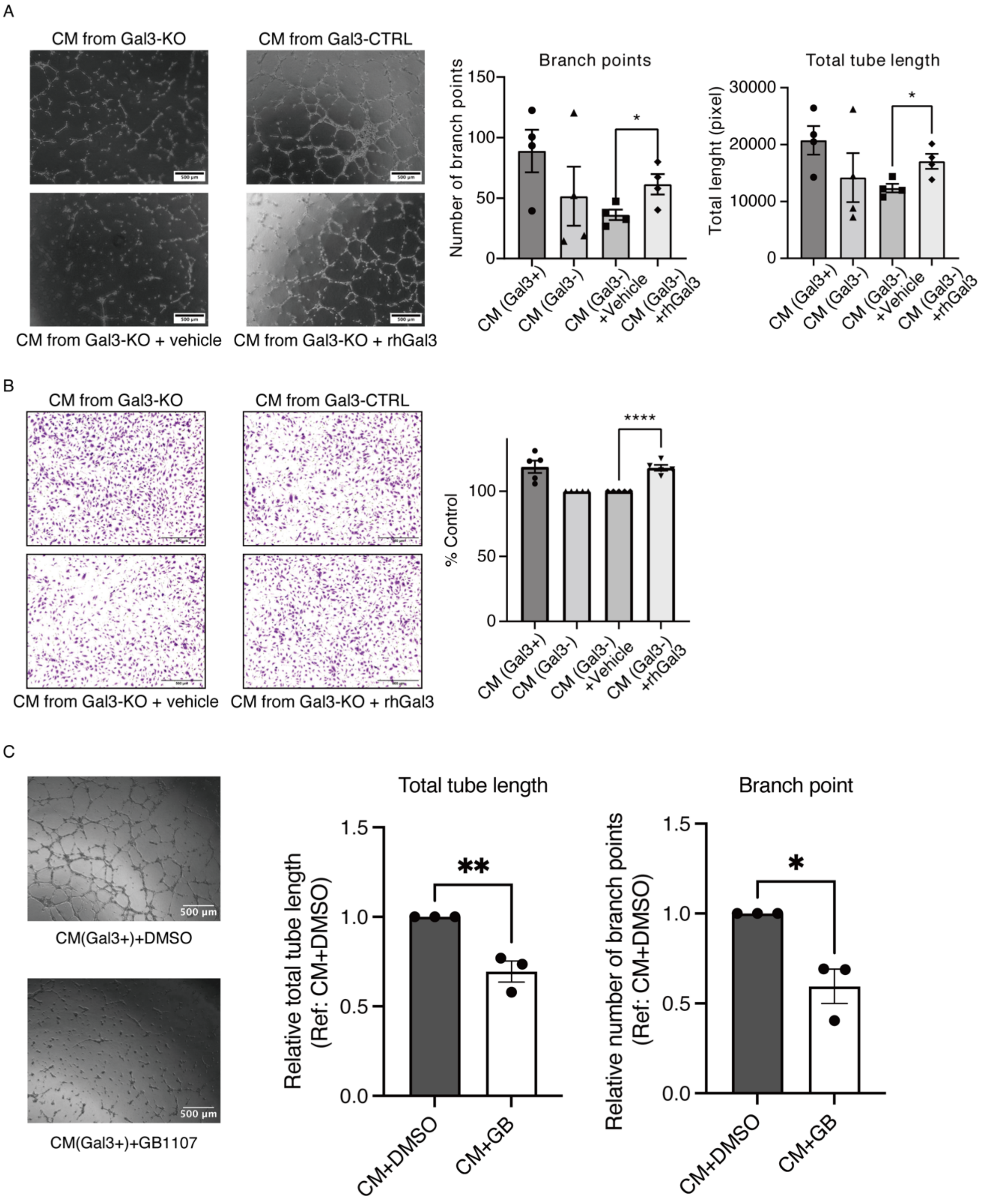

Supplementary Fig. S11: Assays related to the angiogenesis following the addition of CM, rhGal3, and/or Gal3-SMI. A) Tube forming assay on HUVECs following the addition of CM and rhGal3 or vehicle into Matrigel layer. B) Angiogenesis invasion assay on HUVECs following the addition of CM and rhGal3 or vehicle into culture media. C) Tube forming assay on HUVECs following the addition of CM and GB1107 or vehicle into Matrigel layer. Significance was calculated using t-test with * indicating $P < 0.05$, ** indicating $P < 0.01$, *** indicating $P < 0.001$, and **** indicating $P < 0.0001$.

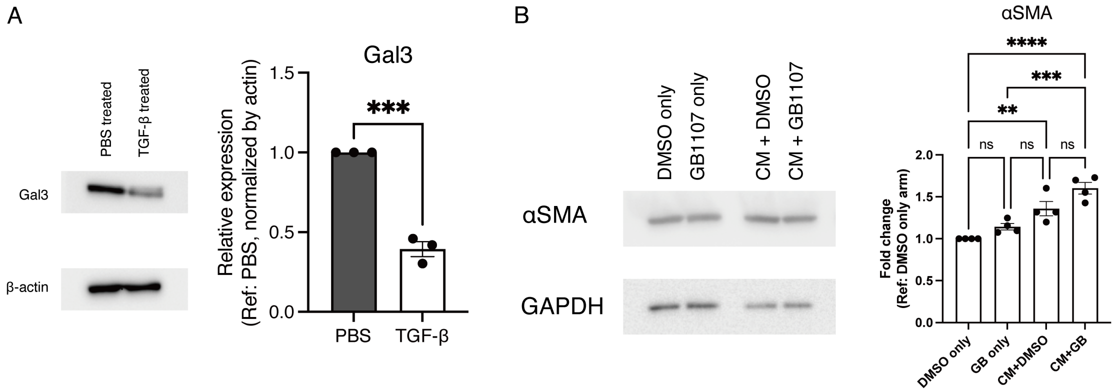

Supplementary Fig. S12: Gal3 expression in fibroblasts and the effect of Gal3-SMI on αSMA expression in fibroblasts. A) Immunoblotting result for Gal3 in fibroblast exposed to PBS (vehicle) or TGF-β for 48 hours. Significance was calculated using t-test with *** indicating P < 0.001. B) αSMA expression in fibroblasts exposed to GB1107 or Gal3-positive CM + GB1107. No significant difference was obtained between DMSO only and GB1107 only arm and between CM+DMSO and CM+GB1107 arm. Significance was calculated using Tukey's multiple comparisons test with ** indicating P < 0.01, *** indicating P < 0.001, and **** indicating P < 0.0001.

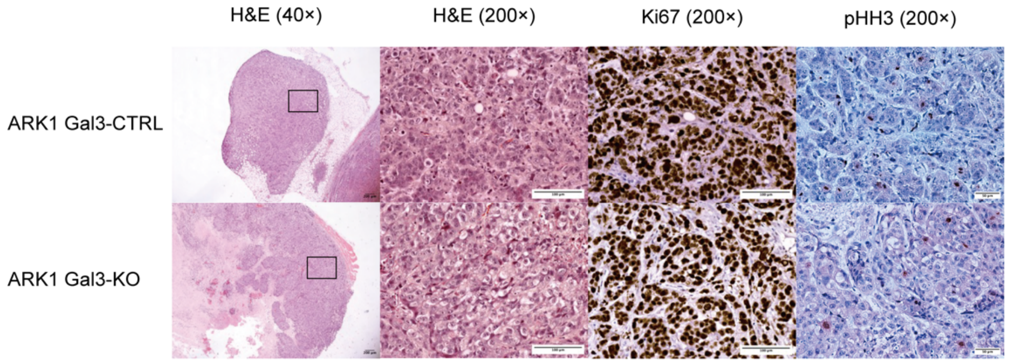

Supplementary Fig. S13: H&E and IHC results for Ki67 and phospho-Histone H3 in tumor sections in the subcutaneous model.

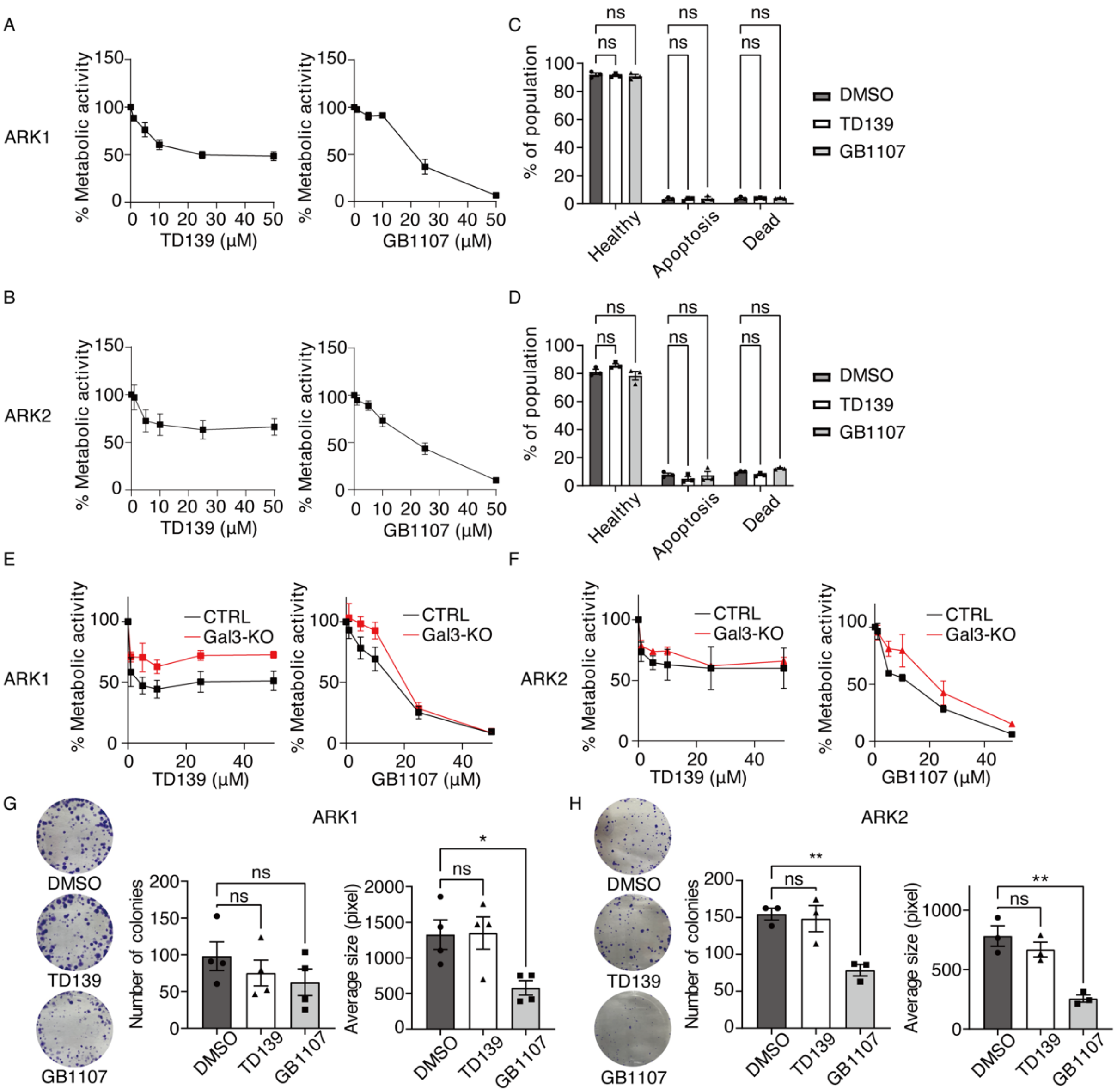

Supplementary Fig. S14: Testing Gal3-SMIs (TD139 and GB1107). A) MTT assay in ARK1 cells exposed with TD139/GB1107 for 72 hours. B) MTT assay in ARK2 cells exposed with TD139/GB1107 for 72 hours. C) Cell death analysis using flow cytometry in ARK1 cells exposed with TD139/GB1107. D) Cell death analysis using flow cytometry in ARK2 cells exposed with TD139/GB1107. E) MTT assay using TD139/GB1107 in ARK1 Gal3-CTRL and Gal3-KO cells to assess the off-target effects. F) MTT assay using TD139/GB1107 in ARK2 Gal3-CTRL and Gal3-KO cells to assess the off-target effects G) Colony forming assay in ARK1 cells exposed to the Gal3-SMIs. H) Colony forming assay in ARK2 cells exposed to Gal3-SMIs. Significance was calculated using Dunnett's test with * indicating $P < 0.05$, ** indicating $P < 0.01$, *** indicating $P < 0.001$, and **** indicating $P < 0.0001$.

Supplementary Detailed Methodology

Lists of abbreviations/antibodies/reagents/equipment/searched are provided separately.

To investigate the relationship between Gal3 expression and clinical features

The Cancer Genome Atlas (TCGA) database analysis: The expression levels of LGALS3 and their correlation with progression-free survival (PFS) and overall survival (OS) were evaluated using the TCGA database through the website (https://www.cbioportal.org/). The TCGA Pan-cancer atlas was utilized as the database. Patients exhibiting LGALS3 expression z-scores > than 1 were categorized as belonging to the LGALS3 high group, while those with LGALS3 expression z-scores < than -1 were assigned to the LGALS3 low group. A comparison was made between the LGALS3 high and low groups for patients with endometrioid carcinoma and uterine serous cancer (USC), respectively.

Clinical samples: Paraffin blocks representing primary surgical cases whereby the patients had a diagnosis of uterine serous cancer were retrieved from either the Massachusetts General Hospital (MGH) Department of Pathology archives or from the Vincent Center for Reproductive Biology (VCRB)- MGH Gyn Repository. All samples were obtained under Institutional Review Board approved protocol (MGB022P003117 and DFHCC 07-049). Patient derived USC organoids were generated from samples originally obtained by the VCRB banking personnel after gaining informed under the approved protocol (DFHCC 07-049) or provided by Dr. Sarah Hill, Dana Farber Cancer Institute.

Evaluation of galectin 3 expression in primary tumors and its relationship to clinic-pathological features: All formalin-fixed paraffin-embedded (FFPE) tumor samples and their clinical correlate

information were collected under an approved IRB (MGB022P003117 and DFHCC 07-049). Formalin-fixed paraffin-embedded tissue representing the primary tumor of from each patient. All patients were confirmed to have uterine serous cancer. The FFPE blocks were sliced to a thickness of 5μm using a microtome, and glass slides were prepared. Tissue sections were subjected to hematoxylin and eosin (H&E) staining and Gal3 immunohistochemistry (IHC) using the same method described before(1). Anti-Gal3 goat polyclonal IgG (R&D Systems, Minneapolis, MN, USA, catalog number: AF1197, 1:100) was validated using Gal3 positive cells and Gal3 knockout (Gal3-KO) cell line (supplementary Fig. S1A-B). Two certified pathologists blinded to the original pathology report and diagnosis, scored Gal3 expression by the percentage of Gal3-positive tumor cells as previously reported(2). The clinical information collected included age at diagnosis, body mass index (BMI), stage, degree of myometrium invasion (<1/2 or ≥1/2), lymphovascular space invasion (LVSI), cervical involvement, serosal invasion, presence of extrauterine lesions (ovarian, omentum, vaginal, parametrium, pelvic or para-aortic lymph nodes, other organs).

Cell lines and organoids culture

Established human, USC cell lines were provided by Dr. A. Santin (Yale University, New Haven, CT, USA) and have been previously characterized (3, 4). ARK1 and ARK2 cells were cultured in the complete media; RPMI1640 medium (Life Technologies, Grand Island, NY, USA, catalog number: 11875093) supplemented with 10% fetal bovine serum (FBS) (Life Technologies, catalog number: 26140079) and 1% penicillin- streptomycin (Life Technologies, catalog number: 15140122). As previously described(1), we detected amplification of HER2 (ERBB2) in both cell lines and a PIK3CA mutation (E542K) in ARK1. SKOV3 cell, an ovarian

cancer cell line, was maintained as we described previously(5). Human umbilical vein endothelial cells (HUVECs) were purchased from LONZA (Lonza, Walkersville, MD, USA, catalog number: C2519A) and were maintained in EBM-2 medium (Lonza, catalog number: CC-3156) supplemented with Endothelial Cell Growth Medium 2 Supplement Pack (Lonza, catalog number: C-39211). IMR90 cells, normal fibroblast cells, were kindly provided by Dr. Cesar Castro (Massachusetts General Hospital, Boston, MA, USA). This cell line was maintained in Eagle's Minimum Essential Medium (EMEM) (ATCC, Manassas, VA, USA, catalog number: 30-2003) supplemented with 10% FBS and 1% penicillin-streptomycin. All cells were maintained at 37 °C at 5% CO_2. The cell lines were confirmed negative for mycoplasma prior to any assay by the MycoAlert Mycoplasma Detection Kit (Lonza, catalog number: LT07-318).

The patient derived organoids were maintained as previously described(6).

Generation of Gal3-KO Cells and Knockdown Cells

Oligonucleotides targeting LGALS3 were synthesized, annealed and ligated into the single guide RNA scaffold of the LentiCRISPRv2 (AddGene, Watertown, MA, USA, catalog number: 52961) via the BsmBI sites, according to the method previously described(7). Two different oligo pairs were chosen for construct synthesis targeting the following sequences in exon 3 (sgRNA1) or 4 (sgRNA2) of LGALS3: sgRNA1 GTCTACCCAGGGCCACCCAG and sgRNA2 GCTGATAACAATTCTGGGCA.

Successful construct generation was verified by Sanger sequencing of the sgRNA region. Transfections of ARK1 and ARK2 lines were performed using Lipofectamine 2000 (Invitrogen, Carlsbad, CA, USA, catalog number: 11668019) with co-transfection of pLenti-C-mGFP-P2A-

Puro Lentiviral Gene Expression Vector (OriGene Technologies, Inc. Rockville, MD, USA, catalog number: PS100093) to detect the cells which were transfected. The GFP positive cells were isolated by flow cytometry (FACSAria™ Fusion Flow Cytometer, BD Life Sciences, San Jose, CA, USA) and single cells were plated in individual well in 96 well plate. Lysates of individual CRISPR-Cas9-edited clones were evaluated by western blot for Gal3 expression. Biallelic knockout through indel generation was confirmed in each line following PCR amplification of genomic DNA extracted using DNeasy Blood & Tissue Kits (QIAGEN, Hilden, Germany, catalog number: 69506) with primers flanking the guide RNA target of the LGALS3 gene (For sgRNA1: forward; CTCAAGGATGGCCTGGCGCATG, reverse; GGTCTCCCTGAAGCCCTCTGCA. For sgRNA2: forward; GCCTTATCTCTTTGGCCCCTGGG, reverse; CCTGTTTGCATTGGGCTTCACCG). PCR products were sent to AZENTA for Sanger sequence (South Plainfield, NJ, USA). All primers were synthesized by IDT (Coralville, IA, USA). Gal-3 KO cells obtained using sgRNA1 in ARK1 and sgRNA2 in ARK2 and Gal3 control cells (Gal3-CTRL) generated using the same method without gRNA were used in subsequent experiments.

The SKOV3 Gal3 knockdown (Gal3-KD) cell line was generated utilizing the shRNA system and the materials and methodology previously described(8).

Conditioned Media (CM) for add back experiments.

ARK2 Gal3-CTRL and ARK2 Gal3-KO cells were counted and plated at concentrations estimated to reach 70-80% confluence after three days. The day after plating, the complete media was changed out to media containing 10% or 2% FBS. After 48 hours, the conditioned medium

was collected and centrifuged at 3500 rpm for 5 minutes. The supernatant was filtered with a 0.2 µm surfactant free cellulose acetate (SFCA) filter (Thermo Scientific, Carlsbad, CA, USA, catalog number: 09-740-105) and applied to the further experiments.

Extracellular vesicles (EVs) extraction: EVs were isolated from 10 ml of ARK2 culture media with sub-confluent condition on a 10 cm dish using Amicon® Ultra-15 Centrifugal Filter Unit (Millipore Sigma, Burlington, MA, USA, catalog number: UFC910024). Debris was excluded from the media by centrifugation and a 0.22 µm sterile syringe filter system. Then, the EVs were isolated by centrifugation (at 3,000 × g for 30 min, rinsed with filtered DPBS, and spun at 3,000 × g for 30 min). All procedures were performed at 4°C. The concentrated EVs were diluted with lysis buffer and subjected to sonification and subjected to ELISA.

ELISA assay: Gal3 concentrations in conditioned media and EVs were measured using the Human Galectin-3 Quantikine ELISA Kit (R&D Systems, catalog number: DGAL30), following the manufacture's protocol.

To investigate the distribution of the Galectin 3

Immunocytochemistry (ICC): Ten thousand cells of ARK1 and ARK2 were seeded on Falcon 4-well culture slides (Corning Inc., Kennebunk, ME, catalog number: 354114) in 300 µL of complete media. After overnight incubation, the cells were fixed with 4% paraformaldehyde (PFA) (Biotium, Fremont, CA, USA, catalog number: 22023) at room temperature (RT) for 15 minutes. Subsequently, the cells were rinsed three times with PBS and permeabilized using 0.1% Triton X (Sigma-Aldrich, St. Louis, MO, USA, catalog number: X100), followed by another three rinses. The cells were then incubated with 3% bovine serum albumin (BSA) (Sigma-

Aldrich, catalog number: A3294) at RT for 1 hour. Primary antibodies, Anti-Gal3 goat polyclonal IgG (R&D Systems, catalog number: AF1197, 1:300) or Normal Goat IgG Control (R&D Systems, catalog number: AB-108-c), were added to the wells and incubated at RT for 1 hour. After three rinses with PBS and PBST, the cells were incubated with the appropriate secondary antibody, Donkey Anti-Goat IgG H&L (Alexa Fluor® 488) (Abcam, Cambridge, UK, catalog number: ab150129, 1:200), for 1 hour at RT in the dark. Following three rinses with PBS and PBST, the cells were stained with Phalloidin (Invitrogen, 1:400 Alexa Fluor™ 555 Phalloidin, catalog number: A34055) if required, at RT for 1 hour. The cells were then rinsed twice with PBS and mounted with DAPI (Vector Laboratories, Inc., Burlingame, CA, USA, catalog number: H-1200). Finally, the cells were observed and imaged under a 60x magnification using the FV3000 confocal laser scanning microscope (Olympus, Tokyo, Japan).

Assessment of cell viability and proliferation.

Cell counts: 0.1 x 10^6 cells/well were seeded on the 6-well plates (Corning Inc., catalog number: 3516). Cell counts were determined with a TC20 Automated Cell Counter (Bio-Rad Inc, Hercules, CA, USA) at the indicated time points (time zero = O/N incubation, 24, 48, and 72 hours after O/N incubation) in triplicate. Dead cells were excluded using the trypan blue staining (Life Technologies, catalog number: 15250061).

Cell death assessment: 0.1 x 10^6 cells (ARK1 and ARK2 CTL and Gal3-KO) were seeded per well on 6-well plates (Corning Inc., catalog number: 3516) in the complete media. After 24 and 72 hours, all cells (those that were attached and those in the supernatant) were collected and the baseline level of apoptosis and necrosis were assessed by FITC Annexin V Apoptosis Detection

Kit with 7-AAD (BioLegend, San Diego, CA., catalog number: 640922). At a minimum, twenty thousand cells per technical replicate was evaluated with a flow cytometer (Gallios Flow Cytometer, Beckman-Coulter, France). The results were analyzed using FlowJo™ v10.8 Software (BD Life Sciences).

MTT assay to measure the chemosensitivity: Cells were harvested at sub-confluency and plated in 96-well plates (Corning Inc., catalog number: 3596) at 1,000 cells per well in 100 μL of media per well. After overnight incubation, 150 μL of the complete media containing each concentration (0, 1, 2.5, 5, 10, 20 μM) of carboplatin (Sigma-Aldrich, St. Louis, MO, USA, catalog number: C2538-250MG) was added to each well. Cell viability was assessed by MTT assay 72 hours after adding the media, as previously described(9).

BH3 profiling: To ascertain the degree of involvement of galectin 3 in cell priming towards apoptosis, we conducted BH3 profiling as described previously(10).

Cell cycle assay: Cells were seeded at 0.5 x 10^6 cells per well on the 6 well plate (Corning Inc., catalog number: 3516) and incubated overnight. The cells were rinsed with serum-free medium, and then incubated with serum-free media for 24 hours. After which, the media was removed and the complete media was replaced, and the plates were incubated for an additional 6 hours. The cells were trypsinized, collected and processed using the Cell Cycle Phase Determination Kit (Cayman Chemical Company, Ann Arbor, MI, USA, catalog number: 10009349) as per manufactures instructions and assessed by flow cytometry. At least 20,000 events for each end point measure were evaluated. The FlowJo™ v10.8 Software (BD Life Sciences) cell cycle analysis module (Dean-Jett-Fox model) was used to analyze the percentage of population for each of the subG1, G0/G1 phase, S phase, and G2/M phase.

Investigating the impact of loss/inhibition of Gal3 on EGFR signaling

Wild type ARK1 and ARK2 cells were seeded and cultured in a complete media with 10% FBS. When cells reached sub confluence, the media was replaced with complete media containing vehicle (Dimethyl sulfoxide: DMSO) (Fisher Scientific Co, catalog number: D136-1) or 10 µM of GB1107 (MedChemExpress, Monmouth Junction, NJ, USA, catalog number: HY-114409), a Gal3 small molecule inhibitor (SMI). The cells were then maintained in culture for 12 or 72 hours. Cell lysates were recovered and were utilized for immunoblot analysis.

Assessment of Gal3 on EGF/EGFR signaling: Wild type ARK1 and ARK2 cells were cultured in serum-depleted media for 6 hours. The cells were then treated with 20 ng/ml of EGF (Life Technologies, catalog number: PHG0311) + vehicle or 10 µM of GB1107. After 30 minutes, cells were harvested in a lysate buffer, and immunoblotting was run for analysis of EGFR/pEGFR and ERK1/2/pERK1/2.

Assessment of cancer stem cell properties and cancer stem cell markers.

Colony forming assay: Five hundred cells per well were seeded into 6-well plates (Corning Inc., catalog number: 3516) and the medium was replaced every two days (day 3, day 6). At the end of the culture period the cells were fixed with methanol and stained with 0.5% crystal violet (GFS chemicals, Columbus, OH, USA, catalog number: 31201). Images of each well were captured, and the average size of the colonies and the average number of colonies were determined by Fiji software version 2.3.0(11). To examine the add-back effect of Gal3, the colony-forming assay was also conducted by incorporating CM mixed with complete media in a 1:1 ratio instead of using complete media. To further support Gal3 contributes to colony forming

ability, 10 μM of GB1107 or vehicle (DMSO) was added to the CM from ARK2 Gal3-CTRL cells and the colony-forming assay was conducted in ARK1 and ARK2 Gal3-KO cell lines as described above.

Sphere forming assay: Media for the sphere-forming assay included DMEM/F12 (Life Technologies, catalog number: 11330032), 1% Pen/Strep, 10 ng/mL of bFGF recombinant human protein (Life Technologies, catalog number: 13256-029), 20 ng/mL of EGF (Life Technologies, catalog number: PHG0311), and B27 (Life Technologies, catalog number: 17504044). Cells were collected and resuspended in sphere-forming media. 4×10^4 cells (ARK1) or 2×10^4 cells (ARK2) per well with 2 mL of the media were seeded in low attachment plates (Corning Inc., catalog number: CLS3473). Cells were cultured for 14 days adding 100 μL of the media with growth factors every two days. After the 14-day-incubation, tumor spheres in the wells were imaged under 40× magnification of EVOS M5000 Imaging System (Thermo Fisher Scientific, San Jose, CA, USA). The average number of the tumor spheres was calculated.

Evaluation of cancer stem cell markers: ARK1 and ARK2 Gal3-CTRL and Gal3-KO cells were collected under sub-confluent status (roughly 70 to 80%) to analyze the percent of cells displaying the cancer stem cell markers (CD44, CD117, CD133, and aldehyde dehydrogenase (ALDH) activity). For assessment of CD44, CD117, and CD133 populations in the control and Gal3-KO cells, the isotype cell lines were harvested, rinsed twice with the staining buffer (BioLegend, catalog number: 420201) and stained with anti-CD44 antibody conjugated with APC (BD Biosciences, catalog number: 559942, 2:100), anti-CD117 antibody conjugated with PE (BD Biosciences, catalog number: 555714, 5:100) or anti-CD133 antibody conjugated with PE (Miltenyi Biotec, Bergisch, Gladbach, Germany, catalog number: 130-113-748, 2:100) at 4°C

for 30 minutes. The isotype control for CD44 (BioLegend, catalog number: 401210), CD117 (BioLegend, catalog number 400114), and CD133 (BioLegend, catalog number: 401208) were used as the negative controls. The cells were also stained with DAPI (BioLegend, catalog number: 422801) to distinguish between live dead cells. At least 20,000 live singlet cells per technical replicate were assessed by flow cytometry the results were analyzed using FlowJo™ v10.8 Software (BD Life Sciences). ALDH activity was evaluated by using the ALDEFLUOR Kit (StemCell Technologies, Durham, NC, catalog number: 01700) in accordance with manufacturer's instructions. Briefly, cells were harvested, washed, counted, and suspended in ALDEFLUOR buffer at 1×10^6 cells/mL. Cells were divided into two aliquots and incubated with 1.5 mmol/L ALDEFLUOR substrate in the presence or absence of the ALDH inhibitor N,N-diethylaminobenzaldehyde (DEAB). Following a 45-minute incubation at 37°C, the endpoints were assessed via flow cytometer as described above.

Investigating the impact of Gal3 inhibition on Notch1 signaling: Wild type ARK1 and ARK2 cells were seeded and cultured in the organoid culture condition(6). Cells were cultured in the organoid culture media with 10 µM of GB1107 or vehicle (DMSO) for 7 days changing media every other. After 7-days in culture, the cells were collected, and the lysates were utilized for immunoblot analysis.

Exploring the role of Gal3 in metastatic potential

Fibronectin adhesion assay: The fibronectin-coated 96 well-plate was made by adding 80 µL of fibronectin solution (1 µg/mL, Sigma-Aldrich, catalog number: F0556-100UL) and incubating for 1 hour at RT. After removing the solution, wells were rinsed with PBS three times. Ten thousand of ARK1 and ARK2 Gal3-CTRL and Gal3-KO cells per well were seeded with 100 µL

of the complete media and incubated for 1 hour at 37°C, 5% CO_2. After incubation, wells were rinsed gently with PBS, and cells were fixed with methanol for 15 minutes at RT. Cells were then stained with crystal violet for 20 minutes at RT. After staining, the wells were washed with dH_2O, and an image recorded of the areas at the center of each well using a EVOS M5000 Imaging System (Thermo Fisher Scientific) at 40× magnification. The number of adherent cells was counted by Fiji software version 2.3.0(11). To confirm if the pharmacologic inhibition has a similar effect as Gal3-KO in cell adhesion ability, wild type ARK1 and ARK2 cells were cultured in the media containing 10 μM of GB1107 or vehicle (DMSO) for 72 hours (changed media every other day). Then, cells were collected and submitted to the fibronectin adhesion assay, where DMSO or 10 μM of GB1107 were added to the media.

Trans-well migration assay: After a 24 hour-culture in non-serum containing medium, the cells were trypsinized and resuspended in serum-free medium. In each insert, 1×10^5 cells of ARK1 cells in 200 μL of serum-free medium or 0.5×10^5 cells of ARK2 were placed in the upper chamber (Corning Inc., catalog number: 353097) and 500 μL of the complete medium with 10% FBS was added to the lower chamber as a chemoattractant. Cells that migrated across the bottom membrane of the insert (Corning Inc., catalog number: 3470) were fixed with methanol and stained with crystal violet staining solution (GFS chemicals Columbus, catalog number: 31201) after 8 hours (ARK1) and 24 hours (ARK2) incubation. Four images were captured at distinct positions on each insert membrane using Nikon ECLIPSE Ni-U (Nikon, Tokyo, Japan) with a magnification of 40×. The Fiji software version 2.3.0 was employed to quantify the number of migrated cells(11). To confirm if the pharmacologic inhibition has a similar effect as Gal3-KO in cell migration ability, wild type ARK1 and ARK2 cells were cultured in the media containing 10 μM of GB1107 or vehicle (DMSO) for 72 hours (changed media every other day). Then, the

wild type ARK1 and ARK2 cells were collected and submitted to the transwell migration assay, where DMSO or 10 μM of GB1107 were added to the media.

Invasion assay: Corning BioCoat Matrigel Invasion Chambers with 8.0 μm PET membrane 24-well plates were used to determine Gal3 influence on tumor cell invasion (Corning Inc., catalog number: 354480). After 24 hour-culture with non-serum medium, the cells were trypsinized and resuspended in serum-free medium. In each insert, 1×10^5 cells in 200 μL of serum-free medium were placed in the upper chamber and 500 μL of the complete medium with FBS was added to the lower chamber as a chemoattractant. Cells that invaded and migrated across the Matrigel™ layer were imaged and analyzed as described above. To confirm if the pharmacologic inhibition has a similar effect as Gal3-KO in cell invasion ability, wild type ARK1 and ARK2 cells were cultured in the media containing 10 μM of GB1107 or vehicle (DMSO) for 72 hours (changed media every other day). Then, cells were collected and submitted to the Matrigel invasion assay, where DMSO or 10 μM of GB1107 were added to the media.

Determining the impact of Gal3 positive and negative tumor cell condition medium on tumor microenvironment.

Angiogenesis invasion assay using HUVECs: HUVECs were cultured as described above. Biocoat Matrigel Invasion Chambers (Corning Inc., catalog number: 354480) with an 8.0-μm pore polyester membrane were used. The Matrigel™ in each invasion chamber was primed with 200 μL of CM form ARK2 Gal3-CTRL or CM from ARK2 Gal3-KO for 24 hours at 37°C in a 5% CO_2 incubator. Fifty thousand HUVECs were mixed in 200 μL of endothelial basal culture medium with no growth supplement and plated on top of the primed Matrigel™ after the CM

was aspirated. Subsequently, 500 µL of endothelial culture medium with growth medium supplement was placed in the lower reservoir below the invasion chamber as an attractant. The samples were incubated for 16 hours, cells that invaded and migrated across the Matrigel™ layer were imaged and analyzed as described above.

For the add-back experiment using recombinant human galectin 3 (rhGal3) (R&D Systems, catalog number: 1154-GA-050), 5 µg/mL of rhGal3 diluted with PBS containing 0.1% BSA or PBS containing 0.1%BSA was applied to the insert with 200 µL of 2% FBS CM from ARK2 Gal3-KO.

Tube-forming assay using HUVECs: HUVECs were cultured as described above. Forty-eight well plates (Thermo Fisher Scientific, catalog number: 150687) were used, and Cultrex with reduced growth factor (RGF) and basement membrane extract (BME) (R&D Systems, catalog number: 3536-005-02) mixed with CM form ARK2 Gal3-CTRL or ARK2 Gal3-KO (1:1 ratio) were plated in each experimental well. Fifty thousand HUVECs were seeded on top of the RGF BME and incubated at 37 °C, 5% CO_2 for 6 hours. Tubes were assessed at 6 hours across all conditions with the EVOS M5000 Imaging System (Thermo Fisher Scientific) and quantified with Wimasis image analysis software (https://www.wimasis.com/en/WimTube). Four representative images were taken per well under 40× magnification. Total tube length and number of branch points were used as the endpoints.

For the add-back experiment using rhGal3 (R&D Systems, catalog number: 1154-GA-050), 5 µg/mL of rhGal3 diluted with PBS containing 0.1% BSA or PBS containing 0.1% BSA was applied to the BME mixed with CM from Gal3-KO and run the same assay.

To further support the impact of Gal3 in tube forming, 10 μM of GB1107 or vehicle (DMSO) was applied to the BME mixed with CM from ARK2 Gal3-CTRL cells and the tube forming assay was conducted as described above.

Assessment of Gal3 influence on the transition of fibroblasts to cancer associated fibroblast (CAFs): IMR90 cells were seeded on Falcon 4 well culture slide (Corning Inc., catalog number: 354114) with 10,000 cells per chamber for ICC or on a Falcon 6 cm dish (Corning Inc., catalog number 353002) with 150,000 cells per dish. After overnight incubation, cells were treated with a 1:1 mixture of the complete media described above and 10% CM (from ARK2 Gal3-CTRL or ARK2 Gal3-KO). After 48 hours of incubation, treated cells were either assessed via ICC or lysates were subjected to western blot. A positive control included cells treated with 10 ng/mL of Transforming Growth Factor-β1 (TGF-β1) (PeproTech, Rocky Hill, NJ, USA, catalog number: 100-21) and the negative controls was vehicle (PBS) alone. ICC was conducted using the same method described before. The Human/Mouse/Rat alpha-Smooth Muscle Actin Antibody (R&D Systems, catalog number: MAB1420) was employed as the primary antibody at a dilution of 1:100, while the same concentration of the Mouse IgG2A Isotype Control (R&D Systems, catalog number: MAB003) was utilized as the isotype control. The F(ab')2-Goat anti-Mouse IgG (H+L) Cross-Adsorbed Secondary Antibody (Invitrogen, catalog number: A-11017) was employed as the secondary antibody at a dilution of 1:1000. Phalloidin was not utilized in this assay.

After fibroblasts were found to be Gal3-positive, we exposed them to 10 ng/ml TGF-β1 for 48 hours which resulted in reduced Gal3 expression compared with those exposed with vehicle as evidenced by immunoblotting. We then investigated the impact of pharmacological inhibition of Gal3 in fibroblasts and any exogenous Gal3 in CM. To accomplish this, fibroblasts were cultured

in complete media with DMSO (vehicle) or 10 μM GB1107, or 1:1 mixed media with Gal3-positive CM with DMSO (vehicle) or 10 μM GB1107. After 48 hours the expression level of αSMA was assessed by immunoblotting as the indicator of conversion to CAF phenotype.

Confirmation of loss of Gal3 effect using in vivo models and patient derived organoids

Animal protocols: Animal procedures were approved by the Massachusetts General Hospital Institution Animal Care and Use Committee and were performed according to the National Institutes of Health (NIH) Guide for the Care and Use of Laboratory Animals (Protocol number: 2017N000236). Female NSG (NOD scid gamma) mouse, 6 – 8 weeks age, were obtained from Steele Laboratories at MGH. The number of mice used in our in vivo experiments was based on preliminary experiments conducted using these specific cell lines.The experiments did not require blinding and randomization.

In vivo limiting dilution tumorigenic assay: ARK1 Gal3-CTRL and Gal3-KO cells were collected, counted, aliquoted and resuspended in 1:1 PBS: Cultrex Basement Membrane Extract, Type 2 (R&D Systems, catalog number: 3532-010-02) (100 μL of PBS: 100 μL of Cultrex Basement Membrane Extract) solution. Then, 1×10^4, 5×10^4, 1×10^5, and 2×10^5 cells were injected subcutaneously in the dorsal flank of each mouse using 27-gauge needle. Each dilution group had four mice. Mice were palpated for tumor every two days starting on day 3 post injection to determine time to onset. Once tumors were evident, the date was noted, and mice were weighed and palpated twice a week to monitor tumor growth. Mice were to be euthanized when the tumors reached >15 mm or if the mouse lost more than 15% bodyweight or appeared in distress as indicated by ruffled fur or hunched posture or difficulty moving around the cage.

Mice were euthanized in accordance with our IACUC protocol. After euthanizing the mice, tumors were collected, weighed and processed for further investigation.

Intraperitoneal tumor implantation model: The intraperitoneal (IP) orthotopic model was incorporated to better appreciate the impact of loss of Gal3 on tumor cell implantation, establishment, and potential growth of 1 million of ARK1 Gal3-CTRL or ARK1 Gal3-KO cells injected in a volume of 200 μL PBS into the peritoneal cavity. Sixty days post injection the mice were euthanized. The peritoneal cavity was imaged to document intraperitoneal disseminated lesions, the lesions were harvested, and total tumor weight was determined.

H&E staining and IHC: Xenograft tumor samples were processed, embedded in paraffin and 5 μm sections were prepared. Slides were subjected to H&E, and IHC for Ki67 and phospho-Histone H3 (pHH3). Ki67 (1:100, DAKO, Carpinteria, CA, USA, catalog number: M7240) immunostaining was performed as previously described(1). IHC for pHH3 (1:100, Cell Signaling Technology, Danvers, MA, USA, catalog number: 9701) was performed following the manufacturer's instructions. Simultaneously, staining without primary antibodies was performed (Supplementary Fig. S1C-D. Briefly, after antigen unmasking with 10 mM sodium citrate buffer and quenching the endogenous peroxidase with 3% hydrogen peroxide (Fisher Scientific Co, Pittsburgh, PA, USA, catalog number: H325) in dH_2O, nonspecific background was blocked with 6% BSA in 0.1% PBS-T. Primary antibody was diluted in 6% BSA and incubated 1 hour at RT. After washes, sections were incubated with secondary antibody (anti-rabbit IgG conjugated with HRP, 1:50, Santa Cruz Biotechnology, Dallas, TX, USA, catalog number: sc-2357), washed and incubated with a DAB substrate (DAKO, catalog number: SK-4100) and counterstained with hematoxylin.

Confirmation of the effects of Galectin 3 small-molecule inhibitors: The impact of Gal3 small-molecule inhibitors (SMIs), specifically TD139 (Selleck Chemicals, Houston, TX, USA, catalog number: S0471) and GB1107, on cell viability was assessed through MTT and cell death analysis. MTT assay was performed using the previously described methodology, investigating the effects of TD139 and GB1107 at concentrations of 0, 1, 5, 10, 25, and 50 μM for 72-hour incubation.

For cell death analysis, a concentration of 10 μM of each Gal3-SMI was utilized, followed by a 72-hour incubation period and subsequent analysis same as described previously.

The off-target effects of these Gal3-SMIs were confirmed by conducting a similar MTT assay on both Gal3-CTRL and Gal3-KO cells of ARK1 and ARK2.

Colony formation assay was performed in the presence or absence of 10 μM of each Gal3-SMI for 72 hours. The culture medium containing Gal3-SMIs was changed out every other day. The other procedure was same as described.

Investigation of Gal3 pharmaceutical inhibition effect using patient-derived organoids: Organoids were cultivated on the 48-well plate (Corning Inc., catalog number: 3548) using the methodology explicated above. The organoids were seeded in Matrigel (Corning Inc., 356231) and subsequently cultured in the organoid culture medium, which contained 10 μM of GB1107 or the vehicle (DMSO). The media was replenished every other day, and the entire area occupied by the organoids was quantified observed using an Incucyte Live Cell System and Incucyte's automated Organoid Software Analysis Module (Essen BioScience, Ann Arbor, MI, USA).

Immunoblot analysis

Immunoblot analysis was performed as previously described(1). Antibodies directed against Galectin 3 (R&D Systems, catalog number: AF1197), Cyclin A2 (Cell Signaling Technology, catalog number: 4656), Cyclin B1 (Cell Signaling Technology, catalog number: 4138), Cdk1 (Cell Signaling Technology, catalog number: 9116), Cdk2 (Cell Signaling Technology, catalog number: 2546), Notch1 (Cell Signaling Technology, catalog number: 3608), HES1 (Cell Signaling Technology, catalog number: 11988), HEY1 (Proteintech, Rosemont, IL, USA, catalog number: 19929-1-AP), αSMA (R&D Systems, catalog number: MAB1420), EGFR (Cell Signaling Technology, catalog number: 4267), phospho-EGFR (Cell Signaling Technology, catalog number: 3777), ERK (Cell Signaling Technology, catalog number: 4695), phospho-ERK (Cell Signaling Technology, catalog number: 9106), GAPDH (Cell Signaling Technology, catalog number: 5174), and β-actin (Cell Signaling Technology, catalog number: 8457) were used. The dilution for all primary antibodies was 1:1000 except for Galectin 3 (1:2000). After incubation with the primary antibody, blots were incubated with the corresponding secondary antibody at RT for 1 hour. Then blots were developed using a chemiluminescent detection reagent (ECL reagent. Prometheus ProSignal, Genesee Scientific, San Diego, CA, USA, catalog number: 20-301B or Clarity Max™ Western ECL Substrate: Bio Rad Inc, catalog number: 1705062). Each blot was stripped using Re-Blot Plus Strong Antibody Stripping Solution (Millipore Sigma, catalog number: 2504) when the same membrane was used to reprobe with another primary antibody. Images were acquired using Bio Rad Chemi Doc Imaging System (Bio-Rad Inc) and analyzed using Bio Rad Image Lab Software (ver.6.1.0) (Bio-Rad Inc).

Data presentation and statistical analysis

All experiments were independently replicated at least three times. Data shown in graph format represent means ± SEM of combined results from all experimental replicates, whereas representative histological photomicrographs are indicated where appropriate. Statistical analyses were done with GraphPad Prism version 9.3.1. T-test was used for experiments in which one outcome or multiple independent outcomes were obtained. One-way ANOVA (analysis of variance) was employed for the comparison among multiple groups, followed by post hoc analysis using the Dunnett's test. Two-way ANOVA was used for the experiments with multiple related outcomes, and group comparisons were performed using Šidák correction. The χ-square test was employed to analyze the contingency table. All statistical analyses were two-sided test. A p-value < 0.05 was considered statistically significant.

References:

1. Groeneweg JW, Hernandez SF, Byron VF, DiGloria CM, Lopez H, Scialabba V, et al. Dual HER2 targeting impedes growth of HER2 gene-amplified uterine serous carcinoma xenografts. *Clin Cancer Res.* 2014;20(24):6517-28.
2. Sumana BS, Shashidhar S, and Shivarudrappa AS. Galectin-3 Immunohistochemical Expression in Thyroid Neoplasms. *J Clin Diagn Res.* 2015;9(11):Ec07-11.
3. English DP, Bellone S, Cocco E, Bortolomai I, Pecorelli S, Lopez S, et al. Oncogenic PIK3CA gene mutations and HER2/neu gene amplifications determine the sensitivity of uterine serous carcinoma cell lines to GDC-0980, a selective inhibitor of Class I PI3 kinase and mTOR kinase (TORC1/2). *Am J Obstet Gynecol.* 2013;209(5):465.e1-9.

4. English DP, Roque DM, Carrara L, Lopez S, Bellone S, Cocco E, et al. HER2/neu gene amplification determines the sensitivity of uterine serous carcinoma cell lines to AZD8055, a novel dual mTORC1/2 inhibitor. *Gynecol Oncol.* 2013;131(3):753-8.
5. Garrett LA, Growdon WB, Rueda BR, and Foster R. Influence of a novel histone deacetylase inhibitor panobinostat (LBH589) on the growth of ovarian cancer. *J Ovarian Res.* 2016;9(1):58.
6. Hill SJ, Decker B, Roberts EA, Horowitz NS, Muto MG, Worley MJ, Jr., et al. Prediction of DNA Repair Inhibitor Response in Short-Term Patient-Derived Ovarian Cancer Organoids. *Cancer Discov.* 2018;8(11):1404-21.
7. Sanjana NE, Shalem O, and Zhang F. Improved vectors and genome-wide libraries for CRISPR screening. *Nat Methods.* 2014;11(8):783-4.
8. Rao TD, Fernández-Tejada A, Axelrod A, Rosales N, Yan X, Thapi S, et al. Antibodies Against Specific MUC16 Glycosylation Sites Inhibit Ovarian Cancer Growth. *ACS Chem Biol.* 2017;12(8):2085-96.
9. van Meerloo J, Kaspers GJ, and Cloos J. Cell sensitivity assays: the MTT assay. *Methods Mol Biol.* 2011;731:237-45.
10. Fraser C, Ryan J, and Sarosiek K. BH3 Profiling: A Functional Assay to Measure Apoptotic Priming and Dependencies. *Methods Mol Biol.* 2019;1877:61-76.
11. Schindelin J, Arganda-Carreras I, Frise E, Kaynig V, Longair M, Pietzsch T, et al. Fiji: an open-source platform for biological-image analysis. *Nat Methods.* 2012;9(7):676-82.

Abbreviations list

Abbreviations	Full term
7-AAD	7-aminoactinomycin D
αSMA	alpha smooth muscle actin
ALDH	Aldehyde dehydrogenases
ANOVA	Analysis of variance
BCL-2	B-cell-lymphoma-2
BH3	Bcl-2 homology domain 3
BID	BH3-interacting domain death agonist
BIM	Bcl-2–interacting mediator of cell death
BSA	Bovine Serum Albumin
Cas9	CRISPR-associated protein 9
CD117	Cluster of Differentiation 117
CD133	Cluster of Differentiation 133
CD44	Cluster of Differentiation 44
CM	Conditioned media
CRDs	Carbohydrate recognition domains
CRSPR	Clustered regularly interspaced short palindromic repeats
CSCs	Cancer stem cells
DAB	3,3′-Diaminobenzidine
DAPI	4',6-diamidino-2-phenylindole
DMSO	Dimethyl sulfoxide
EGF	Epidermal growth factor
EGFR	Epidermal growth factor receptor
EMEM	Eagle's Minimum Essential Medium
ERK1/2	Extracellular signal-regulated kinase 1/2
FBS	Fetal bovine serum
FIGO	International Federation of Gynecology and Obstetrics
FITC	Fluorescein isothiocyanate
Gal3	Galectin 3
Gal3-CTRL	Gal3 control
Gal3-KD	Gal3 knockdown
Gal3-KO	Gal3 knockout
H&E	Hematoxylin and eosin
HUVECs	Human umbilical vein endothelial cells
ICC	Immunocytochemistry
IHC	Immunohistochemistry
IP	Intraperitoneal
LVSI	lymphovascular space invasion

MGH	Massachusetts General Hospital
MTT	3-(4,5-Dimethylthiazol-2-yl)-2,5-diphenyltetrazolium bromide
MUC16	Mucin 16
MW	molecular weight
NIH	National Institutes of Health
NSG mice	NOD.Cg-Prkdcscid Il2rgtm1Wjl/SzJ mice
OS	Overall survival
PBS	Phosphate buffered saline
PFS	Progression-free survival
pHH3	Phospho-Histone H3
rhGal3	recombinant human galectin 3
RNA	Ribonucleic acid
RPMI	Roswell Park Memorial Institute Medium
RT	Room temperature
SEM	Standard error of the mean
SFCA	surfactant free cellulose acetate
sgRNA	single guide RNA
SMI	Small-Molecule Inhibitor
TCGA	The Cancer Genome Atlas
TGF-β	Transforming growth factor beta
TME	Tumor microenvironment
USC	Uterine serous carcinoma
VCRB	Vincent Center for Reproductive Biology

Antibodies list

Application	Antigen	Primary/Secondary	Host	mono/poly	Company	Address	Cat #	dulation	Loading protein (μg/well)	ECL	Note
WB	Human/Mouse/Rat Galectin-3 Antibody	Primary	Goat	poly	R&D Systems	Minneapolis, MN, USA	AF1197	1:2000	15	normal	10% Gel
	Human/Mouse/Rat alpha -Smooth Muscle Actin Antibody	Primary	mouse	mono	R&D Systems	Minneapolis, MN, USA	MAB1420	1:1000	15	normal	10% Gel
	Cyclin A2 (BF683) Mouse mAb	Primary	mouse	mono	Cell Signaling Technology	Danvers, MA, USA	4656	1:1000	15	high sensitivity	10% Gel
	Cyclin B1 Antibody	Primary	rabbit	poly	Cell Signaling Technology	Danvers, MA, USA	4138	1:1000	15	high sensitivity	10% Gel
	cdc2 (POH1) Mouse mAb	Primary	mouse	mono	Cell Signaling Technology	Danvers, MA, USA	9116	1:1000	15	normal	10% Gel
	CDK2 (78B2) Rabbit mAb	Primary	Rabbit	mono	Cell Signaling Technology	Danvers, MA, USA	2546	1:1000	15	normal	10% Gel
	Notch1 (D1E11) XP® Rabbit mAb	Primary	Rabbit	mono	Cell Signaling Technology	Danvers, MA, USA	3608	1:1000	whole: 50<br>cleaved: 30	whole: high<br>cleaved: normal	Whole Notch: 3-8% Gel<br>Cleaced Notch: 4-12% Gel
	HES1 (D6P2U) Rabbit mAb #11988	Primary	Rabbit	mono	Cell Signaling Technology	Danvers, MA, USA	11988	1:1000	30	high sensitivity	4-12% Gel
	HEY1 Polyclonal antibody	Primary	Rabbit	poly	Proteintech	Rosemont, IL, USA	19929-1-AP	1:1000	30	high sensitivity	4-12% Gel
	EGF Receptor (D38B1) XP® Rabbit mAb	Primary	Rabbit	mono	Cell Signaling Technology	Danvers, MA, USA	4267	1:1000	20	normal	4-12% Gel
	Phospho-EGF Receptor (Tyr1068) (D7A5) XP® Rabbit mAb	Primary	Rabbit	mono	Cell Signaling Technology	Danvers, MA, USA	3777	1:1000	20	normal	4-12% Gel
	p44/42 MAPK (Erk1/2) (137F5) Rabbit mAb	Primary	Rabbit	mono	Cell Signaling Technology	Danvers, MA, USA	4695	1:1000	20	normal	4-12% Gel
	Phospho-p44/42 MAPK (Erk1/2) (Thr202/Tyr204) (E10) Mouse mAb	Primary	Rabbit	mono	Cell Signaling Technology	Danvers, MA, USA	9106	1:1000	20	normal	4-12% Gel
	β-Actin (D6A8) Rabbit mAb	Primary	Rabbit	mono	Cell Signaling Technology	Danvers, MA, USA	8457	1:1000	Loading control	normal	Loading control
	GAPDH (D16H11) XP® Rabbit mAb	Primary	Rabbit	mono	Cell Signaling Technology	Danvers, MA, USA	5174	1:1000	Loading control	normal	Loading control
	Anti-mouse IgG, HRP-linked Antibody	Secondary	Horse		Cell Signaling Technology	Danvers, MA, USA	7076	1:10000	NA	NA	
	Anti-rabbit IgG, HRP-linked Antibody	Secondary	Goat		Cell Signaling Technology	Danvers, MA, USA	7074	1:10000	NA	NA	
	Goat IgG HRP-conjugated Antibody	Secondary	Donkey		R&D Systems	Minneapolis, MN, USA	HAF109	1:5000	NA	NA	
ICC	Human/Mouse/Rat Galectin-3 Antibody	Primary	Goat	poly	R&D Systems	Minneapolis, MN, USA	AF1197	1:300	NA	NA	
	Normal Goat IgG Control	Primary	Goat	poly	R&D Systems	Minneapolis, MN, USA	AB-108-c	see note	NA	NA	Apply same concentration as primary antibody
	Alexa Fluor™ 555 Phalloidin	Primary	NA	NA	Invitrogen	Carlsbad, CA, USA	A34055	1:400	NA	NA	
	Human/Mouse/Rat alpha -Smooth Muscle Actin Antibody	Primary	mouse	mono	R&D Systems	Minneapolis, MN, USA	MAB1420	1:100	NA	NA	
	Mouse IgG2A Isotype Control	Primary	mouse	mono	R&D Systems	Minneapolis, MN, USA	MAB003	see note	NA	NA	Apply same concentration as primary antibody
	Donkey Anti-Goat IgG H&L	Secondary	Donkey		Abcam	Cambridge, UK	ab150129	1:200	NA	NA	Alexa Fluor® 488
	F(ab')2-Goat anti-Mouse IgG (H+L) Cross-Adsorbed Secondary Antibody	Secondary	Goat		Invitrogen	Carlsbad, CA, USA	A-11017	1:1000	NA	NA	Alexa Fluor™ 488
IHC	monoclonal mouse anti-human Ki-67	Primary	Mouse	mono	Dako	Glostrup, Denmark	M7240	1:100	NA	NA	follow the kit protocol
	Phospho-Histone H3 (Ser10) Antibody	Primary	Rabbit	poly	Cell Signaling Technology	Danvers, MA, USA	9701	1:100	NA	NA	
	β-Catenin (D10A8) XP® Rabbit mAb	Primary	Rabbit	mono	Cell Signaling Technology	Danvers, MA, USA	8480	1:100	NA	NA	
	Rabbit IgG, polyclonal - Isotype Control	Primary	Rabbit	poly	Abcam	Cambridge, UK	ab37415	see note	NA	NA	Apply same concentration as primary antibody
	Human/Mouse/Rat Galectin-3 Antibody	Primary	Goat	poly	R&D Systems	Minneapolis, MN, USA	AF1197	1:100	NA	NA	
	Normal Goat IgG Control	Primary	Goat	poly	R&D Systems	Minneapolis, MN, USA	AB-108-c	see note	NA	NA	Apply same concentration as primary antibody
	mouse anti-rabbit IgG-HRP	Secondary	mouse		Santa Cruz Biotechnology	Dallas, TX, USA	sc-2357	1:50	NA	NA	
	Rabbit Anti-Goat IgG H&L (HRP)	Secondary	Rabbit		Abcam	Cambridge, UK	ab6741	1:1000	NA	NA	
Flow	Annexin V (FITC Annexin V Apoptosis Detection Kit with 7-AAD)	Primary	NA	NA	BioLegend	San Diego, CA, USA	640922	1:100	NA	NA	conjugated with FITC
	7AAD (FITC Annexin V Apoptosis Detection Kit with 7-AAD)	Primary	NA	NA	BioLegend	San Diego, CA, USA	640922	5:100	NA	NA	
	BD Pharmingen™ APC Mouse Anti-Human CD44	Primary	Mouse	IgG2b k	BD Biosciences	San Jose, CA, USA	559942	2:100	NA	NA	conjugated with APC
	BD Pharmingen™ PE Mouse Anti-Human CD117	Primary	Mouse	IgG1 k	BD Biosciences	San Jose, CA, USA	555714	5:100	NA	NA	conjugated with PE
	CD133/2 Antibody, anti-human	Primary	Mouse	IgG2b k	Miltenyi Biotec	Bergisch Gladbach, Germany	130-113-748	2:100	NA	NA	conjugated with PE
	ALDEFLUOR™ Kit	NA	NA	NA	StemCell Technologies	Durham, NC, USA	1700	see note	NA	NA	Kit
	Cell Cycle Phase Determination Kit	NA	NA	NA	Cayman Chemical Company	Ann Arbor, MI, USA	10009349	see note	NA	NA	Kit
	APC Mouse IgG2b, κ Isotype Ctrl Antibody	Primary	mouse	IgG2b k	BioLegend	San Diego, CA, USA	401210	see note	NA	NA	Apply same concentration as primary antibody
	PE Mouse IgG1, κ Isotype Ctrl (FC) Antibody	Primary	Mouse	IgG1 k	BioLegend	San Diego, CA, USA	400114	see note	NA	NA	Apply same concentration as primary antibody
	PE Mouse IgG2b, κ Isotype Ctrl Antibody	Primary	mouse	IgG2b k	BioLegend	San Diego, CA, USA	401208	see note	NA	NA	Apply same concentration as primary antibody
	DAPI (4',6-Diamidino-2-Phenylindole, Dilactate)	NA	NA	NA	BioLegend	San Diego, CA, USA	422801	1:100	NA	NA	1mg/ml

Reagent/equipment/website list

Reagent/equipment/website	Company	Address	Catalog number	Apprication
6 cm dish	Corning Inc.	Kennebunk, ME, USA	353002	cell culture
10 cm dish	Corning Inc.	Kennebunk, ME, USA	351029	cell culture
6-well plate	Corning Inc.	Kennebunk, ME, USA,	3516	cell culture
12-well plate	Life Technologies	Grand Island, NY, USA	150628	cell culture
24-well plate	Corning Inc.	Kennebunk, ME, USA	3524	cell culture
48-well plate	Thermo Fisher Scientific	San Jose, CA, USA	150687	cell culture
48-well plate (for organoids)	Corning Inc.	Kennebunk, ME, USA	3548	cell culture
96-well plate	Corning Inc.	Kennebunk, ME, USA,	3596	cell culture
Human umbilical vein endothelial cells	Lonza	Walkersville, MD, USA	C2519A	cell culture
EBM-2 medium	Lonza	Walkersville, MD, USA	CC-3156	cell culture
Endothelial Cell Growth Medium 2 Supplement Pack	Lonza	Walkersville, MD, USA	C-39211	cell culture
RPMI 1640 Medium	Life Technologies	Grand Island, NY, USA	11875093	cell culture (ARK1, ARK2, SKOV3)
Eagle's Minimum Essential Medium (EMEM)	ATCC	Manassas, VA, USA	30-2003	cell culture (IMR90)
Fetal Bovine Serum, qualified	Life Technologies	Grand Island, NY, USA	26140-079	cell culture (except HUVECs)
penicillin- streptomycin	Life Technologies	Grand Island, NY, USA	15140122	cell culture
PBS, pH 7.4	Life Technologies	Grand Island, NY, USA	10010023	cell culture
MycoAlert Mycoplasma Detection Kit	Lonza	Walkersville, MD, USA	LT07-318	cell culture
Trypan Blue Solution, 0.4%	Life Technologies	Grand Island, NY, USA	15250061	cell culture
Trypsin-EDTA (0.25%), phenol red	Life Technologies	Grand Island, NY, USA	25200056	cell culture
Thermo Scientific™ Nalgene™ Sterile Syringe Filters	Thermo Scientific	Carlsbad, CA, USA	09-740-105	conditioned medium
Corning® Matrigel® Matrix, Phenol Red Free, Corning®, Matrigel Matrix GFR	Corning Inc.	Kennebunk, ME, USA	356231	Organoid culture
GlutaMAX™ Supplement	Life Technologies	Grand Island, NY, USA	35050061	Organoid culture
HEPES (1M)	Life Technologies	Grand Island, NY, USA	15630080	Organoid culture
Recombinant Human R-Spondin-1	PeproTech	Rocky Hill, NJ, USA	120-38	Organoid culture
Recombinant Human Noggin	PeproTech	Rocky Hill, NJ, USA	120-10C	Organoid culture
Animal-Free Recombinant Human EGF	PeproTech	Rocky Hill, NJ, USA	AF-100-15	Organoid culture
Recombinant Human FGF-10	PeproTech	Rocky Hill, NJ, USA	100-26	Organoid culture
Recombinant Human FGF-basic (154 a.a.)	PeproTech	Rocky Hill, NJ, USA	100-18B	Organoid culture
Nicotinamide,bioreagent, suitable for cell culture, suitable for insect cell culture	Sigma-Aldrich	St. Louis, MO, USA	N0636-100G	Organoid culture
N-Acetyl-L-cysteine	Sigma-Aldrich	St. Louis, MO, USA	A9165-5G	Organoid culture
Prostaglandin E2	Tocris Bioscience	Bristol, UK	2296	Organoid culture
Sb 202190 monohydrochloride hydrate	Sigma-Aldrich	St. Louis, MO, USA	S7076-25MG	Organoid culture
A 83-01	Sigma-Aldrich	St. Louis, MO, USA	SML0788	Organoid culture
LB Agar, powder (Lennox L agar)	Life Technologies	Grand Island, NY, USA	22700025	Plasmid
LB agar (MILLER)	Sigma-Aldrich	St. Louis, MO, USA	22700025	Plasmid
One Shot™ TOP10 Chemically Competent E. coli	Life Technologies	Grand Island, NY, USA	C404010	Plasmid
Ampicillin sodium salt	Sigma-Aldrich	St. Louis, MO, USA	A0166	Plasmid
QIAwave Plasmid Miniprep Kit	Qiagen	Hilden, Germany	27104	Plasmid
Opti-MEM™ I Reduced Serum Medium	Life Technologies	Grand Island, NY, USA	31985062	Gene editing
Polybrene	American Bioanalytical	Natick, MA, USA	AB01643	Gene editing
LentiCRISPRv2	AddGene	Watertown, MA, USA	52961	Gene editing
pLenti-C-mGFP-P2A-Puro Lentiviral Gene Expression Vector	OriGene Technologies, Inc.	Rockville, MD, USA	PS100093	Gene editing
Lipofectamine 2000	Invitrogen	Carlsbad, CA, USA	11668019	Gene editing
DNeasy Blood & Tissue Kit	Qiagen	Hilden, Germany	69504	Gene editing
Falcon® 4-well Culture Slide	Corning Inc.	Kennebunk, ME, USA	354114	ICC
Paraformaldehyde, 4% in PBS	Biotium	Fremont, CA, USA	22023	ICC
Triton™ X-100	Sigma-Aldrich	St. Louis, MO, USA	X100	ICC
VECTASHIELD® Antifade Mounting Medium with DAPI	Vector Laboratories, Inc	Burlingame, CA, USA	H-1200	ICC
Fisherbrand™ Superfrost™ Plus Microscope Slides	Fisher Scientific Co	Pittsburgh, PA, USA	1255015	IHC
Tomo® adhesion slides, clipped corners	Matsunami Glass	Osaka, Japan	TOM-14	IHC
Citrate Buffer, pH 6.0, 10×, Antigen Retriever	Sigma-Aldrich	St. Louis, MO, USA	C9999	IHC
Hydrogen Peroxide	Fisher Scientific Co	Pittsburgh, PA, USA	H325	IHC
M.O.M.® (Mouse on Mouse) Elite® Immunodetection Kit, Peroxidase	Vector Laboratories, Inc	Burlingame, CA, USA	PK-2200	IHC
DAB Substrate Kit, Peroxidase (HRP)	Dako	Glostrup, Denmark	SK-4100	IHC
Fisherbrand™ Cover Glasses: Rectangles	Fisher Scientific Co	Pittsburgh, PA, USA	12545K	ICC/IHC

Bovine serum albumin	Sigma-Aldrich	St. Louis, MO, USA	A3294	ICC/IHC
Human Galectin-3 Quantikine ELISA Kit	R&D Systems	Minneapolis, MN, USA	DGAL30	ELISA
Amicon® Ultra-15 Centrifugal Filter Unit	Millipore Sigma	Burlington, MA, USA	UFC910024	EV isolation
Cell Staining Buffer	BioLegend	San Diego, CA, USA	420201	Flow cytometer
carboplatin	Sigma-Aldrich	St. Louis, MO, USA	C2538-250MG	MTT
RPMI 1640 Medium, no phenol red	Invitrogen	Carlsbad, CA, USA	11835030	MTT
MTT (3-(4,5-Dimethylthiazol-2-yl)-2,5-Diphenyltetrazolium Bromide)	Invitrogen	Carlsbad, CA, USA	M6494	MTT
Human EGF Recombinant Protein	Life Technologies	Grand Island, NY, USA	PHG0311	EGFR signaling
crystal violet	GFS chemicals	Columbus, OH, USA	31201	Colony, Adhesion, Migration, Invasion
Costar® 6-well Clear Flat Bottom Ultra-Low Attachment Multiple Well Plates	Corning Inc.	Kennebunk, ME, USA	3471	Sphere-forming
Advanced DMEM/F-12	Life Technologies	Grand Island, NY, USA	12634010	Sphere-forming
Human FGF-basic (FGF-2/bFGF) Recombinant Protein	Life Technologies	Grand Island, NY, USA	13256-029	Sphere-forming
Human EGF Recombinant Protein	Life Technologies	Grand Island, NY, USA	PHG0311	Sphere-forming
B-27 supplement (50x) without vitamin A	Life Technologies	Grand Island, NY, USA	12587-010	Sphere-forming
Fibronectin solution human fibroblasts	Sigma-Aldrich	St. Louis, MO, USA	F0556-100UL	Fibronectin adhesion
Falcon® Permeable Support for 24-well Plate with 8.0 µm Transparent PET Membrane	Corning Inc.	Kennebunk, ME, USA	353097	Migration
Falcon® 24-well TC-treated Cell Polystyrene Permeable Support Companion Plate, with Lid	Corning Inc.	Kennebunk, ME, USA	353504	Migration
Biocoat Matrigel Invasion Chambers	Corning Inc.	Kennebunk, ME, USA	354480	Invasion
Cultrex with reduced growth factor (RGF) and basement membrane extract	R&D Systems	Minneapolis, MN, USA	3536-005-02	Tube-forming
Recombinant Human Galectin-3 Protein	R&D Systems	Minneapolis, MN, USA	1154-GA	Angiogenesis invasion, Tube-froming
Transforming Growth Factor-β1 (TGF-β1)	PeproTech	Rocky Hill, NJ, USA	100-21	CAFs
Cultrex Basement Membrane Extract, Type 2	R&D Systems	Minneapolis, MN, USA	3532-010-02	In vivo
UltraPure™ DNase/RNase-Free Distilled Water	Life Technologies	Grand Island, NY, USA	10977015	
Dimethyl Sulfoxide	Fisher Scientific Co	Pittsburgh, PA, USA	D136-1	
TD139	Selleck Chemicals	Houston, TX, USA	S0471	
GB1107	MedChemExpress	Monmouth Junction, NJ, USA	HY-114409	Organoid
RIPA Buffer (10X)	Cell Signaling Technology	Danvers, MA, USA	9806	Immunoblot
protein kinase inhibitor	Santa Cruz Biotechnology	Dallas, TX, USA	sc-213277	Immunoblot
Protease Inhibitor Cacktail	Sigma-Aldrich	St. Louis, MO, USA	P8340	Immunoblot
Phosphatase Inhibitor Cocktail 3	Sigma-Aldrich	St. Louis, MO, USA	P0044	Immunoblot
Protein Assay Reagent A	Bio-Rad Inc	Hercules, CA, USA	5000113	Immunoblot
Protein Assay Reagent B	Bio-Rad Inc	Hercules, CA, USA	5000114	Immunoblot
Protein Assay Reagent S	Bio-Rad Inc	Hercules, CA, USA	5000115	Immunoblot
NuPAGE™ LDS Sample Buffer (4X)	Life Technologies	Grand Island, NY, USA	NP0007	Immunoblot
NuPAGE™ Sample Reducing Agent (10X)	Life Technologies	Grand Island, NY, USA	NP0009	Immunoblot
NuPAGE™ 3 to 8%, Tris-Acetate, 1.5 mm, Mini Protein Gels	Life Technologies	Grand Island, NY, USA	EA03785BOX	Immunoblot
NuPAGE™ 4 to 12%, Bis-Tris, 1.5 mm, Mini Protein Gels	Life Technologies	Grand Island, NY, USA	NP0336BOX	Immunoblot
NuPAGE™ 10%, Bis-Tris, 1.5 mm, Mini Protein Gels	Life Technologies	Grand Island, NY, USA	NP0316BOX	Immunoblot
SeeBlue™ Plus2 Pre-stained Protein Standard	Life Technologies	Grand Island, NY, USA	LC5925	Immunoblot
HiMark™ Pre-stained Protein Standard	Life Technologies	Grand Island, NY, USA	LC5699	Immunoblot
Bolt™ Antioxidant	Life Technologies	Grand Island, NY, USA	BT0005	Immunoblot
NuPAGE™ Tris-Acetate SDS Running Buffer (20X)	Life Technologies	Grand Island, NY, USA	LA0041	Immunoblot
NuPAGE™ MOPS SDS Running Buffer (20X)	Life Technologies	Grand Island, NY, USA	NP0001	Immunoblot
Bolt™ Transfer Buffer (20X)	Life Technologies	Grand Island, NY, USA	BT00061	Immunoblot
Blotting-Grade Blocker	Bio-Rad Inc	Hercules, CA, USA	1706404	Immunoblot
Tris Buffered Saline (20X, for Western Blot Washing)	Boston BioProducts	Ashland, MA, USA	BM-301X	Immunoblot
Tween 20, Fisher BioReagents	Fisher Scientific Co	Pittsburgh, PA, USA	BP337-500	Immunoblot
Ponceau S Solution	Boston BioProducts	Ashland, MA, USA	ST-180	Immunoblot
Prometheus Protein Biology Products 20-300B ProSignal® Pico ECL Reagent	Genesee Scientific	San Diego, CA, USA	20-300B	Immunoblot
Clarity Max™ Western ECL Substrate	Bio-Rad Inc	Hercules, CA, USA	1705062	Immunoblot
ReBlot Plus 10X Strong Antibody stripping solution	Millipore	Germany	2504	Immunoblot
TCGA database		https://www.cbioportal.org/		
AZENTA		https://clims4.genewiz.com/RegisterAccount/Login?returnUrl=https://clims4.genewiz.com//CustomerHome/Index		
Wimasis image analysis software		https://www.wimasis.com/en/WimTube		Tube-forming
TC20 Automated Cell Counter	Bio-Rad Inc	Hercules, CA, USA		Cell count
EVOS M5000 Imaging System	Thermo Fisher Scientific	San Jose, CA, USA		Adhesion, Tube-forming
Nikon ECLIPSE Ni-U	Nikon	Tokyo, Japan		Migration, Invasion, IHC
Nikon Eclipse TE2000-S microscope	Nikon	Tokyo, Japan		ICC

FV3000 confocal laser scanning microscope	Olympus	Tokyo, Japan	ICC
FACSAria™ Fusion Flow Cytometer	BD Life Sciences	San Jose, CA, USA	Single cell sorting
Gallios Flow Cytometer (10 colors/3 lasers)	Beckman-Coulter	Brea, CA, USA	Flow cytometer
Bio Rad Chemi Doc Imaging System	Bio-Rad Inc	Hercules, CA, USA	WB
Incucyte Live Cell System and the automated Organoid Software Analysis Module	Essen BioScience	Ann Arbor, MI, USA	Organoid
FlowJo™ v10.8 Software	BD Life Sciences	San Jose, CA, USA	Flow cytometer
Bio Rad Image Lab Software ver.6.1.0	Bio-Rad Inc	Hercules, CA, USA	WB
GraphPad Prism version 9.3.1 for Windows	GraphPad Software	San Diego, CA, USA	Statistics

Uncropped membranes

Full unedited gel for Fig 2E-F and Fig S4 C-D

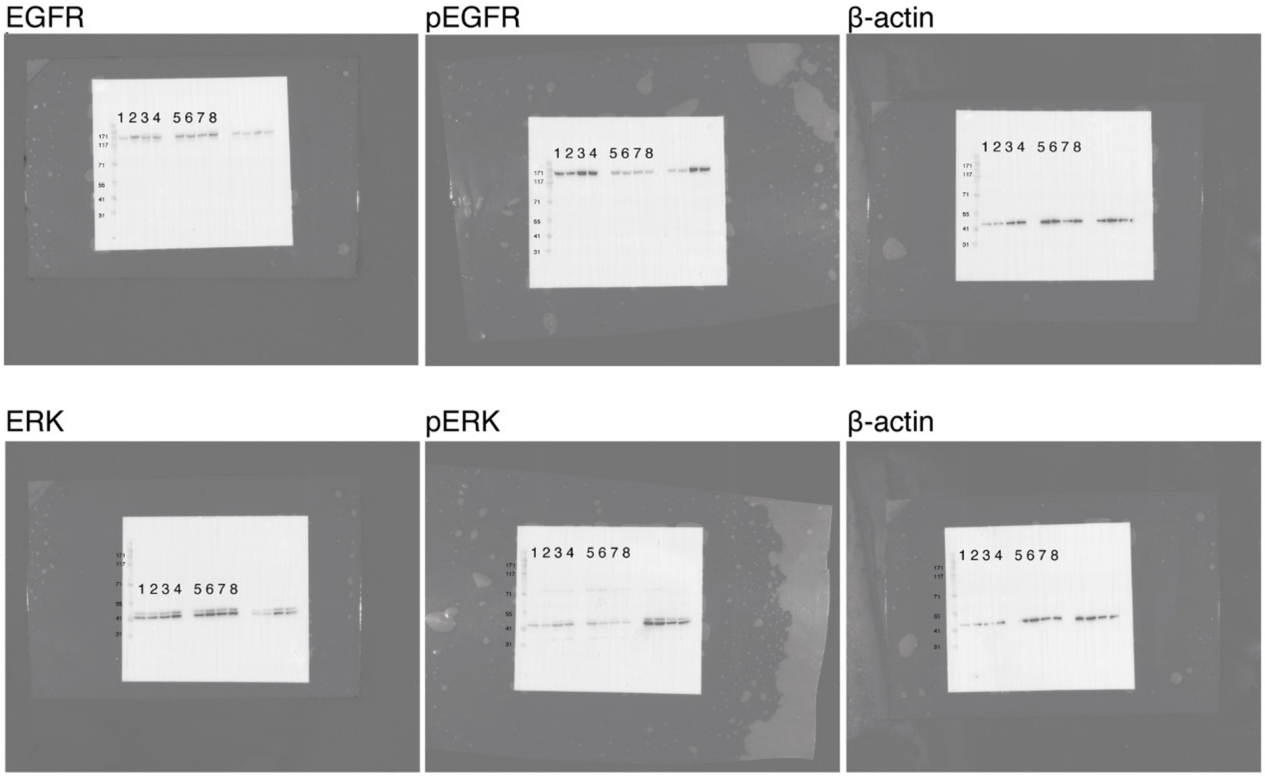

Samples

Lane

1: ARK1 treated with DMSO for 12 hrs

2: ARK1 treated with GB1107 for 12 hrs

3: ARK2 treated with DMSO for 12 hrs

4: ARK2 treated with GB1107 for 12 hrs

5: ARK1 treated with DMSO for 72 hrs

6: ARK1 treated with GB1107 for 72 hrs

7: ARK2 treated with DMSO for 72 hrs

8: ARK2 treated with GB1107 for 72 hrs

Full unedited gel for Fig 3G

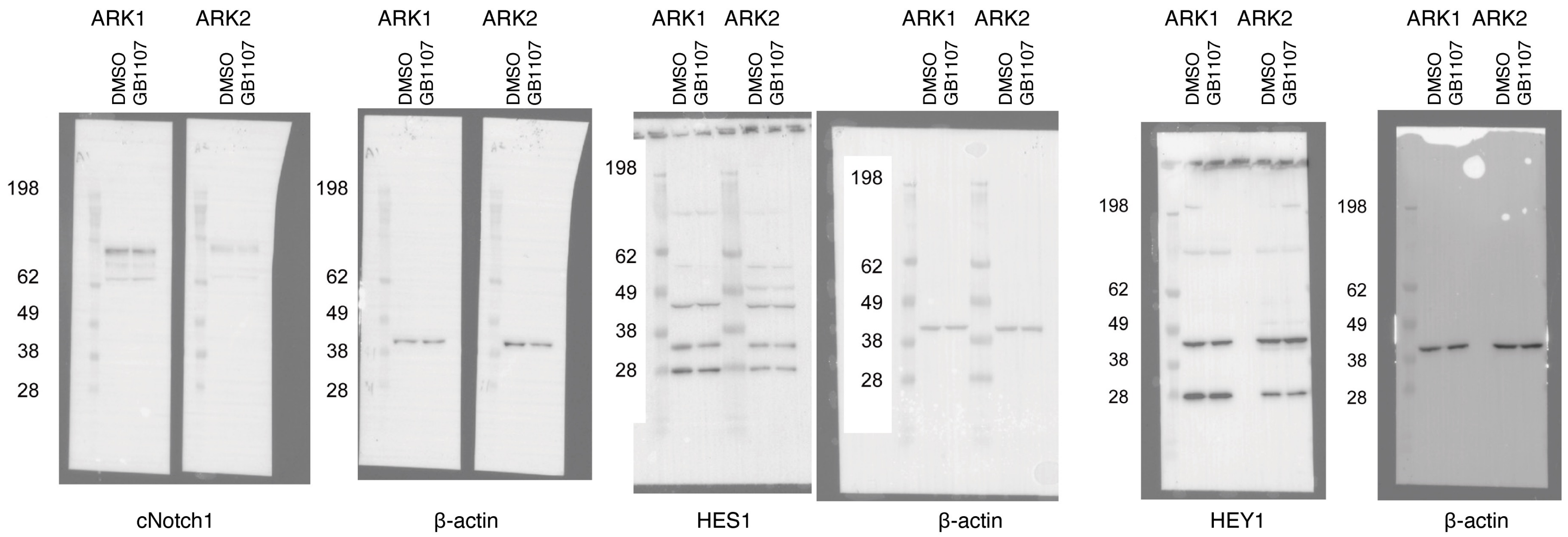

Full unedited gel for Fig 4J

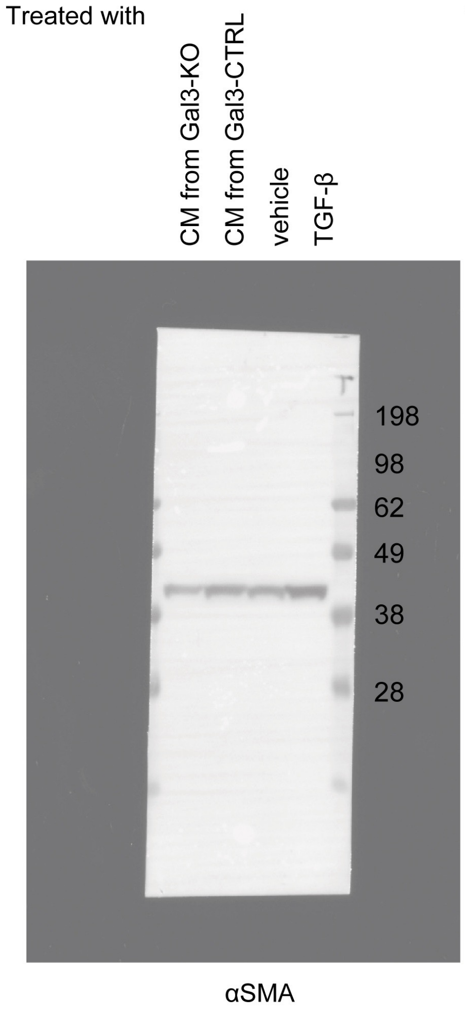

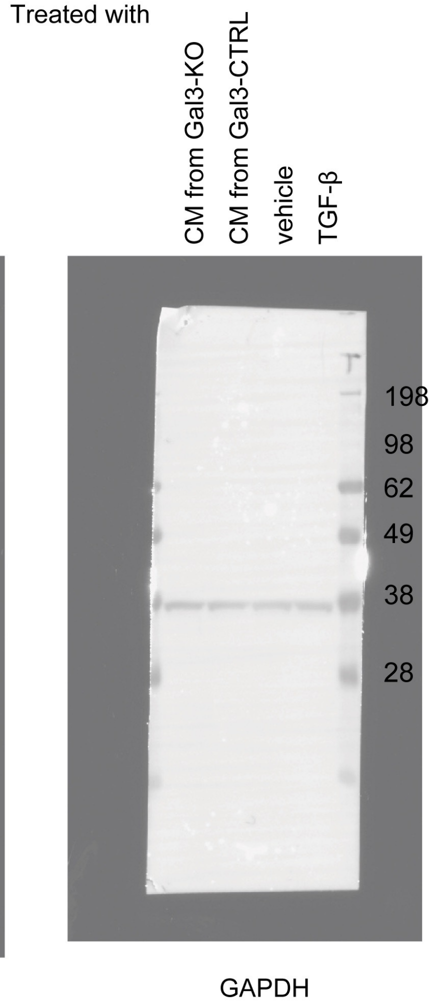

Full unedited gel for Fig S2A

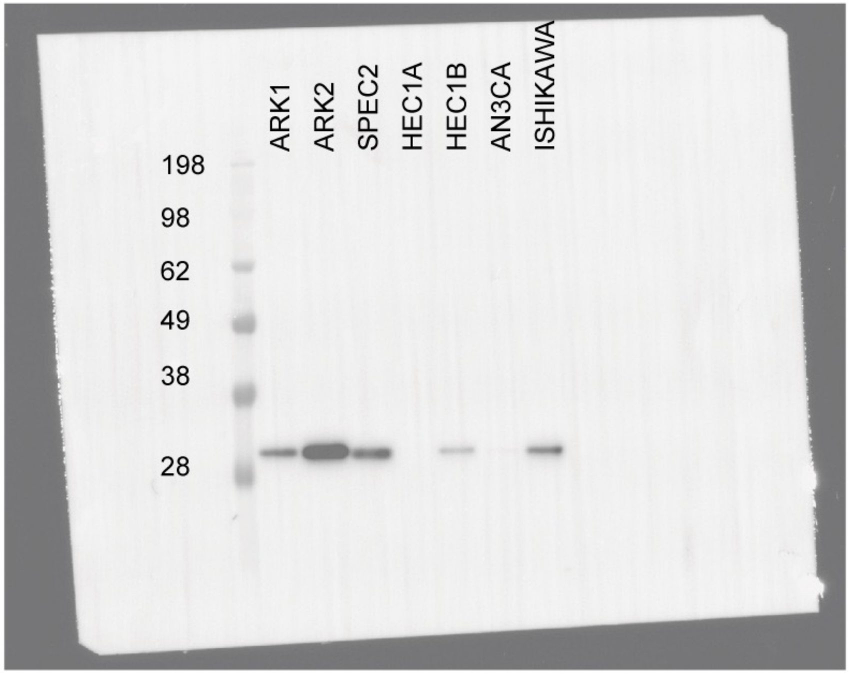

Gal3

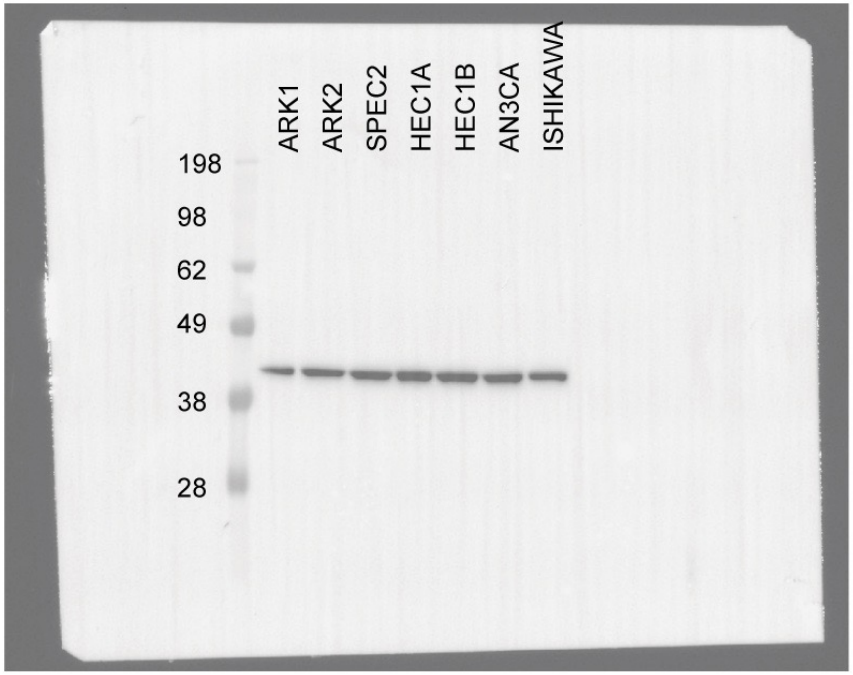

β–actin

Full unedited gel for Fig. S2D

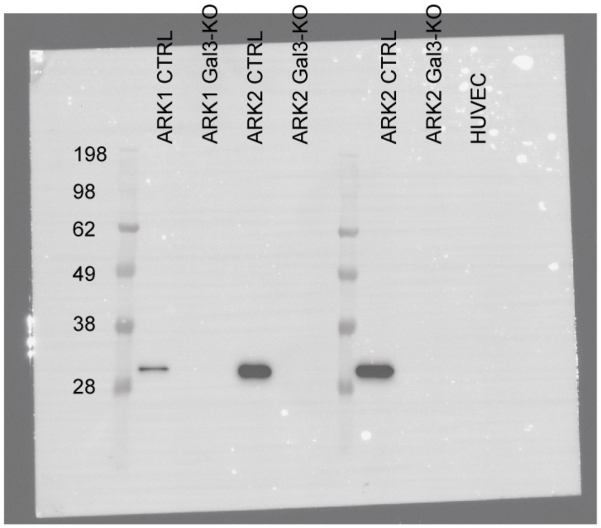

Gal3

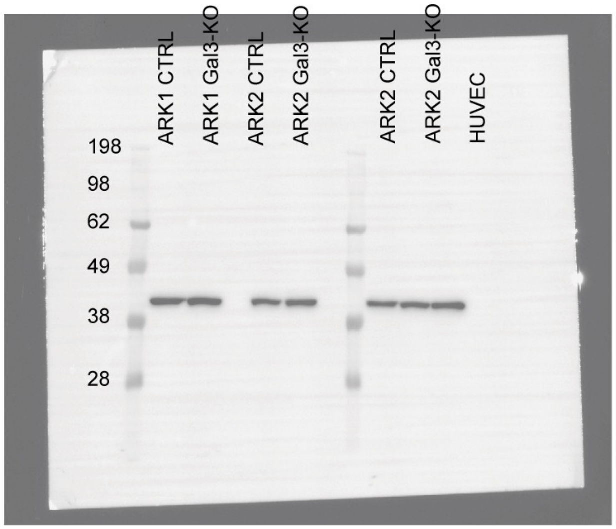

β-actin

Full unedited gel for Fig S3E, F

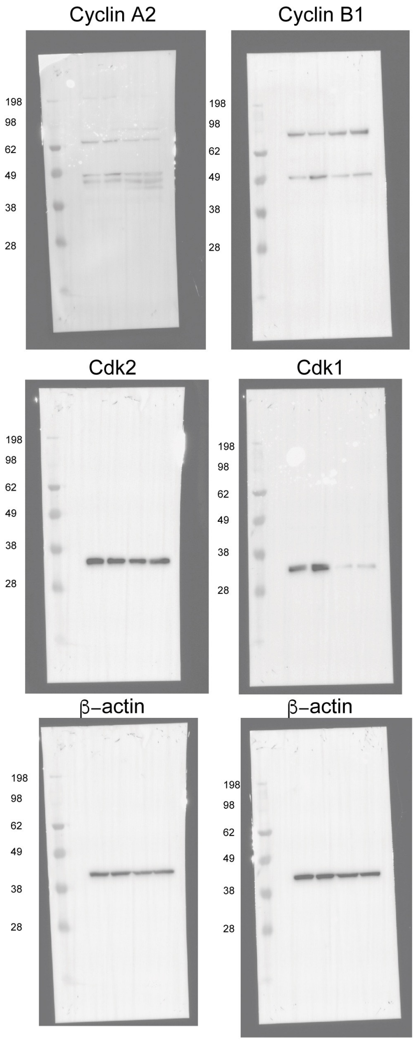

Sample order

From Left

1. ARK1 CTRL
2. ARK1 Gal3-KO
3. ARK2 CTRL
4. ARK2 Gal3-KO

Full unedited gel for Fig S4A-B

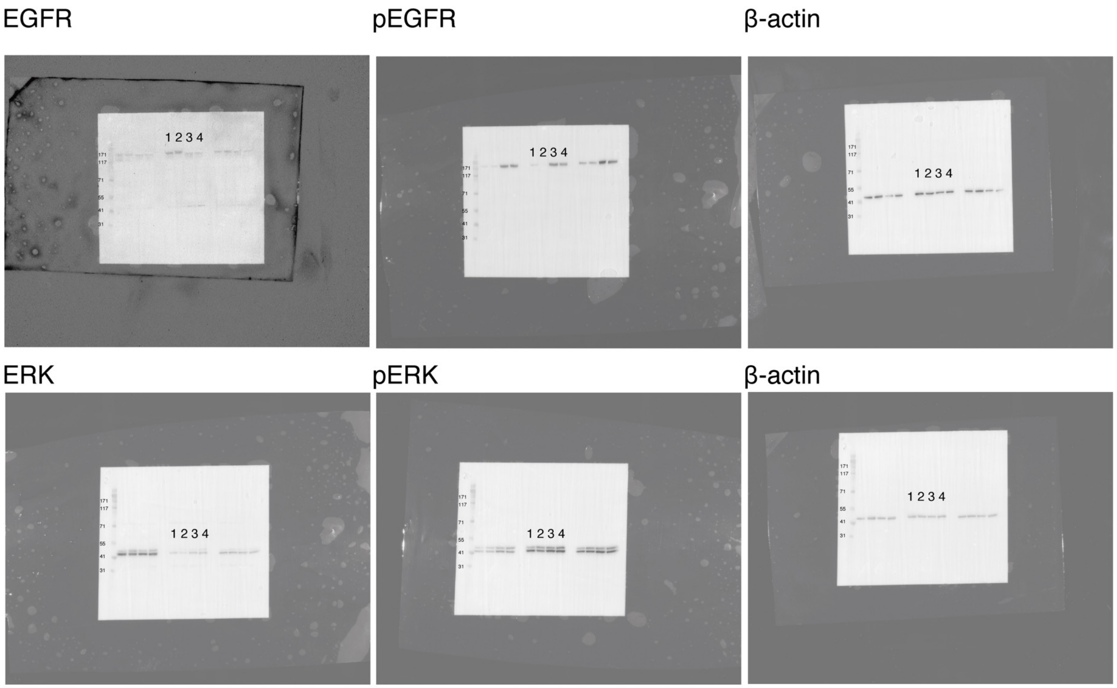

Samples

Lane

1: ARK1 Gal3-CTRL

2: ARK1 Gal3-KO

3: ARK2 Gal3-CTRL

4: ARK2 Gal3-KO

Supplementary Fig. S5

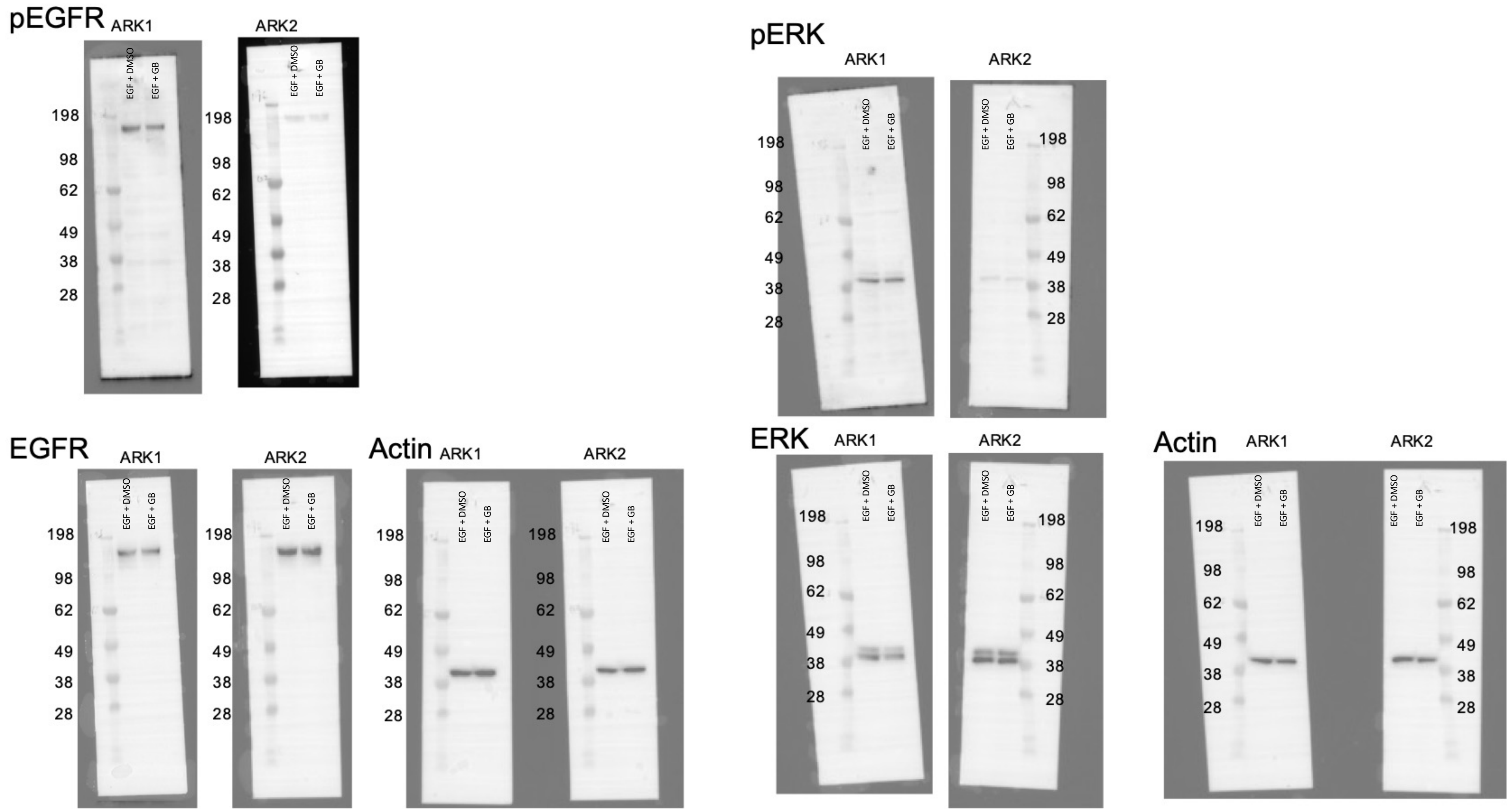

Full unedited gel for Supplementary Fig. S9

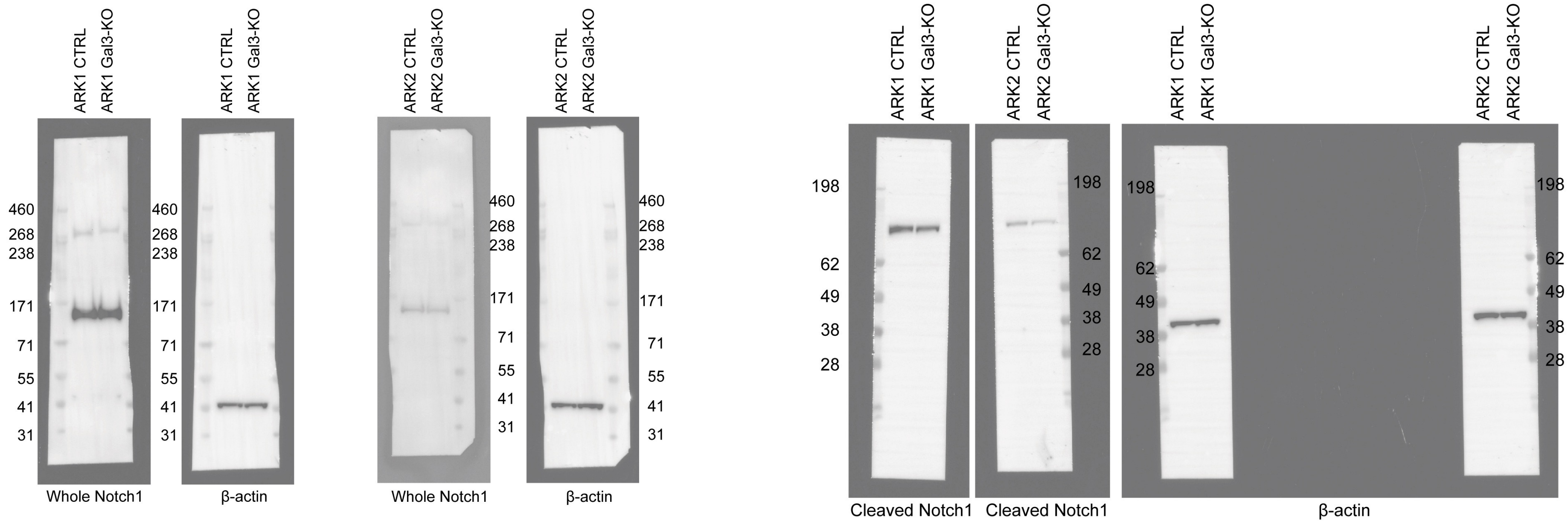

Supplement: Supplementary file 1 — Supplementary data [file 41416_2024_2621_MOESM1_ESM.pdf]
